# Reciprocal nutritional benefits in a Mediterranean seagrass-sponge association

**DOI:** 10.7717/peerj.21392

**Published:** 2026-07-17

**Authors:** Ulisse Cardini, Luis Miguel Montilla, Germán Zapata-Hernández, Johanna Berlinghof, Elisa Guarcini, Marta Furia, Francesca Margiotta, Travis B. Meador, Christian Wild, Simonetta Fraschetti, Irene Olivé

**Affiliations:** 1 Department of Integrative Marine Ecology (EMI), Stazione Zoologica Anton Dohrn, National Institute of Marine Biology, Ecology and Biotechnology, Naples, Italy; 2 Genoa Marine Centre (GMC), Stazione Zoologica Anton Dohrn, National Institute of Marine Biology, Ecology and Biotechnology, Genoa, Italy; 3 Department of Marine Ecology, Faculty for Biology & Chemistry (FB 2), Universität Bremen, Bremen, Germany; 4 Department of Biology, University of Naples Federico II, Naples, Italy; 5 Department of Research Infrastructures for Marine Biological Resources (RIMAR), Stazione Zoologica Anton Dohrn, Naples, Italy; 6 Biology Centre of the Czech Academy of Sciences, České Budějovice, Czech Republic; 7 Faculty of Science, University of South Bohemia, České Budějovice, Czech Republic

**Keywords:** *Posidonia oceanica*, *Chondrilla nucula*, Holobiont, Facilitation, Oxygen fluxes, Nutrient fluxes, Stable isotopes analyses

## Abstract

Sponges commonly form associations within seagrass meadows, but their potential impact on seagrass productivity and nutrient cycles remains poorly understood. This study investigates the association between the demosponge *Chondrilla nucula* and the Mediterranean seagrass *Posidonia oceanica* in two sampling occasions during the plant growth (spring) and senescence (autumn) seasons at a small inlet near Naples, Italy, where the sponge grows conspicuously within the seagrass bed. We found a non-linear relationship between the benthic cover of the sponge and the seagrass, with higher *C. nucula* cover linked to intermediate *P. oceanica* cover, suggesting spatial dependence. *Posidonia oceanica* showed higher net primary production (NPP) in spring, while *C. nucula* was net heterotrophic in spring but exhibited near zero metabolic balance in autumn. NPP remained stable when the two organisms were associated, regardless of the season. *Chondrilla nucula* consistently contributed inorganic nutrients to the association in the form of phosphate, ammonium, and substantial nitrate, recycling nutrients that potentially benefited *P. oceanica* in its growth season. In return, the seagrass released dissolved organic carbon in spring, which is consistent with supporting sponge heterotrophic nutrition. These findings suggest reciprocal benefits in the interaction between *C. nucula* and *P. oceanica*, with nutrient exchange facilitating a facultative mutualism that potentially supports and stabilizes the productivity of the seagrass ecosystem.

## Introduction

Seagrasses are vital ecosystem engineers that create habitats for diverse marine life. Seagrass meadows support significantly more species than unvegetated areas, particularly among fish and invertebrate communities (Heck et al., 2008), while fostering complex epibenthic assemblages (Whippo et al., 2018). Many invertebrates use these meadows as sources of organic matter (Kharlamenko et al., 2001) and as shelter (Boström & Bonsdorff, 1997), and they also host diverse microbiomes that perform key ecosystem functions, contributing to so-called nested ecosystems (Malkin & Cardini, 2021; McFall-Ngai et al., 2013; Pita et al., 2018). Positive species interactions are recognized as crucial drivers of community structure and ecosystem functioning in seagrass ecosystems (Cardini et al., 2019; Cardini et al., 2022; Gagnon et al., 2021; Malkin & Cardini, 2021). However, although this topic has been well explored in terrestrial environments, substantial knowledge gaps remain in marine systems (*e.g.*, Bulleri, 2009).

Among seagrass-associated invertebrates, sponges play a crucial role in nutrient cycling. They consume dissolved organic matter (DOM)—including labile compounds released by primary producers—and rapidly convert it into particulate organic matter through cell turnover, making it available for higher trophic levels through what is known as the sponge loop (De Goeij et al., 2013; Rix et al., 2017). Unlike simple excretion, many sponges mediate complex nitrogen transformations—including nitrogen fixation and nitrification—through their abundant and diverse microbiome, returning inorganic nutrients such as ammonium (${\mathrm{NH}}_{4}^{+}$), nitrate (${\mathrm{NO}}_{3}^{-}$), and phosphate (${\mathrm{PO}}_{4}^{3-}$) to their surroundings and potentially fuelling primary production (Maldonado, Ribes & Van Duyl, 2012). Concurrently, some sponges can shift between net autotrophy—when photosynthesis by their cyanobacterial symbionts exceeds holobiont respiration—and net heterotrophy, when they rely on external organic carbon sources such as DOM to meet their energetic demands (Wilkinson & Trott, 1985; Hudspith et al., 2022). These functions underscore the potential significance of sponges as beneficial partners for primary producers (PP), such as seagrasses and macroalgae, which release large amounts of DOM into their surroundings (Barrón, Apostolaki & Duarte, 2014) and whose growth is often limited by inorganic nitrogen (Touchette & Burkholder, 2000).

Sponge-PP associations have been documented in various marine environments. On coral reefs, sponges absorb DOM from corals and macroalgae, returning inorganic nutrients in a reciprocal, mutually beneficial relationship (Campana et al., 2021; Pawlik & McMurray, 2020; Rix et al., 2017). Similar associations occur in mangrove ecosystems, where sponges release nitrogen that supports mangrove growth, while receiving carbon from mangrove roots, establishing facultative mutualisms (Ellison, Farnsworth & Twilley, 1996).

In seagrass meadows, sponges have been shown to enhance growth and nutrient content of primary producers, as demonstrated in the association of the sponge *Ircina felix* with the seagrass *Thalassia testudinum* and other non-dominant seagrass species (Archer et al., 2021). Another example involves *T. testudinum* benefiting from nutrients like ammonium (${\mathrm{NH}}_{4}^{+}$) and phosphate (${\mathrm{PO}}_{4}^{3-}$) released by the sponge *Halichondria melanadocia* (Archer, Hensel & Layman, 2018; Archer, Stoner & Layman, 2015). However, despite growing research interest in sponge-PP associations, only a limited number of studies have been dedicated to these interactions to date, leaving key ecological processes insufficiently understood. In particular, a quantification of the effect of sponge-seagrass associations on seagrass productivity and nutrient cycles remains largely unexplored, and the extent to which these interactions enhance or stabilize seagrass primary production under varying environmental conditions is still unclear. Addressing these gaps is essential for understanding the resilience of seagrass ecosystems and their capacity to adapt to global change.

In the Mediterranean Sea, the seagrass *Posidonia oceanica* forms extensive meadows that extend from the surface down to about 40 m depth. Among the variety of associated biodiversity, shallow *P. oceanica* meadows frequently host the demosponge *Chondrilla nucula* (Pansini & Pronzato, 1985). The demosponge *C. nucula* is widespread across the Mediterranean Sea and is classified as a high-microbial-abundance (HMA) sponge (Erwin et al., 2012), harboring a rich and diverse microbiome dominated by Cyanobacteria (Mazzella et al., 2024). Similar microbiome compositions have been found in *C. nucula* populations across other Mediterranean locations (Thiel et al., 2007) and in the congeneric *C. caribensis* from the Caribbean (Hill et al., 2006), suggesting stable core bacterial assemblages in *Chondrilla* spp. across regions. Phylogenetic analyses identified cyanobacterial symbionts in *C. nucula* and proposed them as *Candidatus Synechococcus spongiarum* for *C. nucula* from the Mediterranean and *C. australiensis* from Australia (Usher et al., 2004). In the Caribbean species *C. caribensis*, these symbionts provide nutritional benefits through photosynthate translocation and algal cell ingestion (Hudspith et al., 2022).

**Figure 1:**
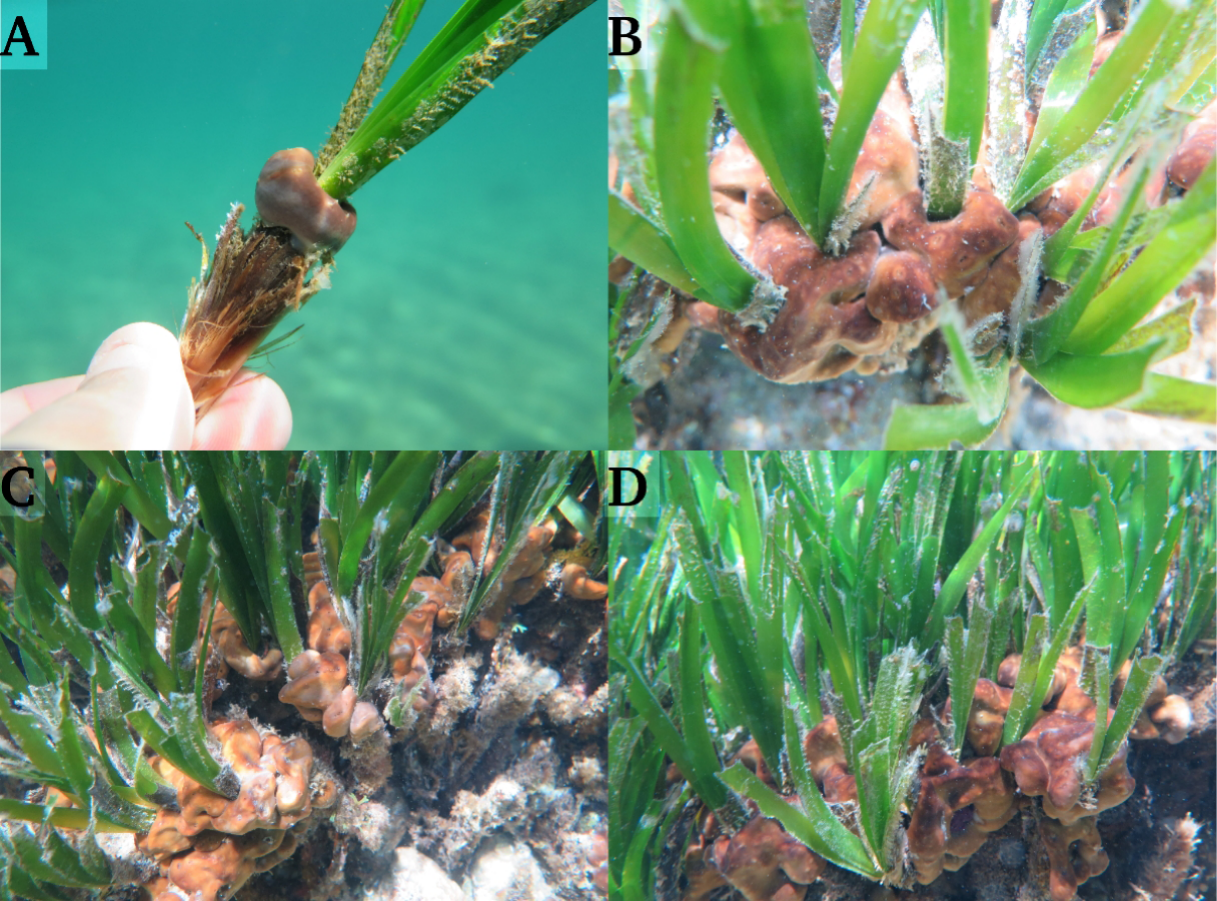
Photographic documentation of the association between *Posidonia oceanica* and *Chondrilla nucula* at the study site. (A) Close-up of a *P. oceanica* shoot with a *C. nucula* bundle surrounding the transition zone between the leaves and foliar sheath. (B) Detail of sponge bundles firmly attached to individual shoots. (C, D) Large *C. nucula* colonies covering multiple shoots and fusing into extensive, contiguous growths. Photos: U. Cardini.

This study investigates the association between *C. nucula* and *P. oceanica* in the central Tyrrhenian Sea (Italy), focusing on a coastal site in the Gulf of Pozzuoli. Here, *C. nucula* grows abundantly at the base of seagrass shoots and expands laterally to adjacent rhizomes, despite the availability of alternative substrates nearby (Fig. 1). We explore whether the *C. nucula-P. oceanica* association can be characterized as a facultative mutualism, hypothesizing that DOM released by *P. oceanica* supports sponge nutrition, while the sponge provides a source of inorganic nutrients to the seagrass.

To test this hypothesis, we: (i) conducted a spatial distribution analysis of *C. nucula* within the seagrass meadow, (ii) quantified net fluxes of oxygen, organic, and inorganic nutrients in closed chamber incubations, and (iii) used stable isotope analyses to examine potential signals of nutrient transfer in the sponge-seagrass association. These experiments evaluated the effect of each organism, both individually and in association, during the plant growth (spring) and senescence (autumn) seasons. Together, these approaches helped to gain clarity on the nature of this sponge-seagrass association and determine whether nutrient exchange plays a role, supporting the characterization of this association as a facultative mutualism.

## Materials & Methods

A preprint version of this manuscript is available (Cardini et al., 2024).

### Study area and benthic cover

Experiments were conducted in the “Schiacchetiello” inlet (40.7938N, 14.0870E, Southern Tyrrhenian Sea, Mediterranean), located in the municipality of Bacoli, Italy. Here, the seagrass *P. oceanica* grows at depths of 0–6 m, forming a patchy meadow. In many patches, the sponge *C. nucula* is found growing at the base of seagrass shoots, enveloping the rhizomes (see Figs. 1A–1D). We conducted video transects by snorkeling parallel to the coastline along the longest possible distance within each shallow (0.5–2 m) seagrass patch in November 2021 (transect lengths: 4.8–11 m). Because the meadow is highly fragmented, sampling was selective rather than systematic, with transects placed to capture benthic cover within individual patches; inter-patch distances ranged from approximately 2 to 20 m. Video footage was processed using FFMPEG (https://ffmpeg.org/) to extract frames, from which 82 photoquadrats were randomly selected from high quality images. A 0.5 m reference measuring bar was included in the footage of the video transects and used to estimate the size of the photoquadrats. We used these photoquadrats to estimate the cover percentage of the primary benthic substrates within replicated 0.25 m^2^ quadrats, resulting in a total surveyed area of 20.5 m^2^. Inorganic and organic nutrient concentrations, as well as light intensity and temperature data, were collected upon each sampling occasion with methods as those reported in the following section to characterize environmental conditions at the study site where sampling took place for the following incubations.

### Oxygen and nutrient fluxes

Shoots of *Posidonia oceanica* (hereafter referred to as ‘seagrass’), *C. nucula* bundles (‘sponge’), and seagrass shoots hosting *C. nucula* (‘association’) were collected from shallow beds (∼1.5 m depth) for *in situ* closed-chamber incubations, as described by Pfister et al. (2023). Briefly, each incubation chamber consisted of a cylindrical transparent polycarbonate container fitted with a gas-tight lid. A single unit—one seagrass shoot, one sponge bundle, or one associated seagrass-sponge unit—was placed inside each chamber together with ambient seawater. We used orthotropic seagrass shoots, complete with leaves and their vertical rhizome, to preserve the shoot’s metabolic autonomy (*e.g.*, Olivé et al., 2022; Olivé et al., 2017). Sponge bundles were handled carefully to minimize disturbance to the animal. Similar-sized organisms and associations were selected for each treatment and allowed for a short recovery period of approximately 30 min before being carefully moved to the chambers, with air contact being avoided. Following the recovery period, only sponge specimens displaying open oscula—indicating active pumping—were used for incubations. These experiments were performed during midday hours on one sampling occasion in each of two seasons: autumn (November 2021) and spring (May 2022). We acknowledge that the lack of within-season replication limits our ability to assess intra-seasonal variability. However, the study was designed to capture distinct differences between two key periods in the plant’s life cycle: the growth season (spring) and the senescence season (autumn). These represent critical phases of biological activity, and the experiments were intended to provide a comparative snapshot of these distinct functional states. In autumn, incubations for each group (*i.e.,* seagrass, sponge and association) were conducted in triplicate (*n* = 3) for both light and dark chambers, using 0.55 L cylindrical chambers for approximately 3.75 h. In spring, each group was incubated in quadruplicate (*n* = 4) in both light and dark, using 1.1 L chambers for approximately 6 h. This was done to account for the increased leaf length at this time of year, while maintaining a similar biomass-to-volume ratio to ensure an adequate analyte signal. Control chambers (*n* = 3 or 4) containing only seawater were included to measure fluxes from the water column community. Dark chambers were wrapped in three layers of black polyethylene to block light and incubated alongside the light chambers. The chambers were held in floating crates to maintain exposure to natural sunlight (for the light chambers) and seawater temperature, while the natural wave action at the shallow study site provided consistent gentle agitation sufficient to prevent thermal and chemical stratification within the chamber volume. This approach, following Olivé et al. (2016) and Pfister et al. (2023), replicates ambient hydrodynamic conditions and avoids the artefacts associated with mechanical stirring in small closed systems. We acknowledge that wave intensity may have varied slightly over the incubation period. HOBO data loggers were used to monitor light intensity and temperature both within the chambers and externally, ensuring conditions resembled those found *in situ* (Table 1). Discrete measurements made in autumn with a LICOR light sensor allowed us to verify that light chambers received light levels well above saturation irradiance (∼ 400 µmol quanta m^−2^ s^−1^). At the beginning of each incubation, ambient seawater samples were collected to establish initial conditions for all analytes. O_2_ concentrations were measured directly inside each chamber at T_0_ and T_end_ using a portable digital meter (WTW Multi 3430 Set K). At the end of each incubation, 30 mL of seawater from each chamber was collected for dissolved organic carbon (DOC) and nitrogen (DON) analysis, and 20 mL was collected for inorganic nutrient measurements (NH_4_^+^, NO_*x*_^−^, PO_4_^3^^−^). Samples were collected with acid-washed syringes and filtered immediately: DOC/DON samples were filtered using pre-combusted GF/F glass microfiber filters (pore size: 0.7 µm), acidified with 80 µL HCl (6 M), and refrigerated at 4 °C until analysis. Inorganic nutrient samples were filtered through 0.22 µm PES membranes, frozen *in situ*, and stored at −20  °C. DOC/DON was analyzed using a TOC-L Analyzer with TN unit (Shimadzu Corporation, Kyoto, Japan), while inorganic nutrients were measured with a continuous flow analyzer (Flowsys; SYSTEA SpA). Sponge and seagrass samples from each chamber were lyophilized for dry weight determination. Hourly flux rates for oxygen and nutrients were calculated as the change in analyte concentrations over incubation time corrected for the signal in the controls and the effective seawater volume in the chamber, standardized to the dry weight of the organisms and expressed in µmol analyte g DW^−1^ h^−1^.

**Table 1:** Environmental conditions at the study site in the two seasons at the time of the incubation experiments. Bold *p* values indicate significant differences between the two seasons in the respective variable (*t*-test).

**Season**	**NH**_**4**_^+^ ** (µM)**	**NO**_**x**_^−^ ** (µM)**	**PO**_**4**_^3−^ ** (µM)**	**DOC** **(µM)**	**DON** **(µM)**	**Light intensity** **(LUX)**	**Temperature** **(°C)**
Autumn	0.98 ± 0.01	0.97 ± 0.08	0.05 ± 0.01	117.3 ± 12.5	6.4 ± 2.9	49,371 ± 42,228	19.5 ± 0.1
Spring	0.39 ± 0.05	0.66 ± 0.03	0.06 ± 0.01	81.9 ± 4.0	6.2 ± 0.2	66,313 ± 34,418	20.2 ± 0.1
*p* value (df)	**0.003** (5)	**0.009** (5)	0.129 (5)	**0.004** (5)	0.943 (5)	0.314 (27)	**<0.001** (27)

### Stable isotope analyses

To examine potential signals of nutrient transfer in the sponge-seagrass association, in spring 2022 we collected independent samples of *P. oceanica* (leaves and associated epiphytes) and *C. nucula*, when growing associated *vs* non-associated, directly from the field rather than from incubation chambers. This approach was chosen to ensure that isotopic signatures reflect genuine *in situ* conditions, as the progressive changes in nutrient concentrations and oxygen levels within closed chambers could introduce artefacts in *δ*^1^^3^C and *δ*^1^^5^N values, and to allow a larger number of spatially independent replicates to be obtained across the meadow. Epiphytes were gently scraped off seagrass leaves and stored in Eppendorf tubes to avoid influencing the bulk stable isotope composition of the seagrass, and to elucidate their potential role in nutrient transfer. All samples (*i.e.,* leaves, epiphytes, and sponges) were placed in acid-washed vials and lyophilized for 48 h. Dried tissues were ground to a fine powder using a tissue lyser, then acid-fumed before being weighed into silver capsules for isotope analysis. Samples were analyzed using a Flash Elemental Analyzer (Thermo Fisher Scientific) equipped with a single reactor (1,020 °C), along with a MAT 253 Plus isotope ratio mass spectrometer (IRMS) interfaced with a Conflo IV system (Thermo Fisher Scientific, Bremen, Germany). The *δ*^1^^3^C and *δ*^1^^5^N values were normalized to Vienna Pee Dee Belemnite and atmospheric air, respectively, after correcting for blanks, ion source linearity, and standardizing against laboratory working standards and an internal peat soil laboratory standard. Precision was typically <0.1‰  for *δ*^1^^3^C and 0.2‰  for *δ*^1^^5^N. The molar C:N ratios (mol:mol) were calculated from C and N weights in the capsules (µg) and based on their respective molecular weights.

### Data analysis

We examined potential asymmetries in the dependence between seagrass and sponge cover using the “qad” package (Griessenberger, Trutschnig & Junker, 2022) and modeled their relationship using a generalized additive model (GAM) with the “mgcv” package (Wood, 2011). Mean differences in environmental conditions between the two seasons were tested using Student’s *t*-tests for the respective variable. Net primary production (NPP) and respiration (R) were determined based on hourly O_2_ fluxes in light and dark incubations, respectively, following Olivé et al. (2016) and Koopmans et al. (2020). To compute integrated rates of gross primary production (GPP = NPP + —R—), daily net community production (NCP = GPP ×12 - —R— ×24), and daily fluxes for each nutrient (Daily Flux = light flux ×12 + dark flux ×12), we generated analytical combinations of the observed values for light and dark fluxes, assuming equal duration of daylight or darkness as in Cardini et al. (2018). Each pair of independent values was combined using the respective formulas to compute the distribution of integrated rates (*n* = 9 for autumn, *n* = 16 for spring). This output provided a comprehensive distribution of the potential outcomes based on the input datasets. Daily rates were expressed in µmol analyte g DW^−1^ d^−1^. NCP is used here in the holobiont sense, reflecting the integrated metabolic balance of each macroorganism together with its entire associated community—including leaf epiphytes and microbial associates in the case of *P. oceanica*, and cyanobacterial photosymbionts and heterotrophic microbial associates in the case of *C. nucula*. One-way permutation-based analyses of variance (PERMANOVA) using Euclidean distance were performed on each response variable (Anderson, 2017) to test the effects of *Community* (seagrass, sponge, association) and *Season* (autumn, spring) on hourly and daily oxygen fluxes as well as daily nutrient fluxes, while separate PERMANOVAs assessed hourly nutrient fluxes with *Condition* (light, dark) as an additional factor. *δ*^1^^3^C and *δ*^1^^5^N values and C:N ratios were tested for differences among *Sample* types (*P. oceanica* leaves, *P. oceanica* epiphytes, *C. nucula*) and *Association* types (associated *vs* non-associated) using one-way PERMANOVA. Results are reported as mean ± SD. All data analyses were performed in R (R Core Team, 2021). To evaluate the reciprocal nutritional benefits between the sponge and the seagrass, we estimated the sponge daily respiratory carbon (C) demand and the seagrass daily nitrogen (N) demand. These estimates were derived from sponge respiration data and plant NCP, respectively, assuming a respiratory/photosynthetic quotient of 1, a 24 h cycle for sponge respiratory C demand, and a 12:12 h light/dark cycle and average plant C:N ratio of 16 for plant N demand. To quantify the contribution of different C sources to the sponge respiratory C demand, we used the daily sponge DOC uptake and GPP rates to calculate the percentage of C demand met by heterotrophic DOC uptake and photoautotrophic C fixation, respectively. The remainder was attributed to particulate organic carbon (POC) uptake *via* filter-feeding. This approach was conservative because sponge DOC uptake was lower than seagrass DOC release, although it is possible that sponge DOC uptake (and its contribution to the sponge respiratory C demand) increased when in association with the seagrass. For the seagrass, we used daily plant ${\mathrm{NH}}_{4}^{+}$ and NO_*x*_ uptake rates to conservatively estimate the percentage of its total daily N demand potentially fulfilled by NH_4_^+^ and NO_*x*_^−^ released by the sponge. The remainder was attributed to other N sources. This approach was also conservative because seagrass uptake rates were lower than sponge release, although plant uptake rates (and their contribution to the plant N demand) may have increased when associated with the sponge.

## Results

### Environmental conditions and seagrass-sponge association patterns

**Figure 2:**
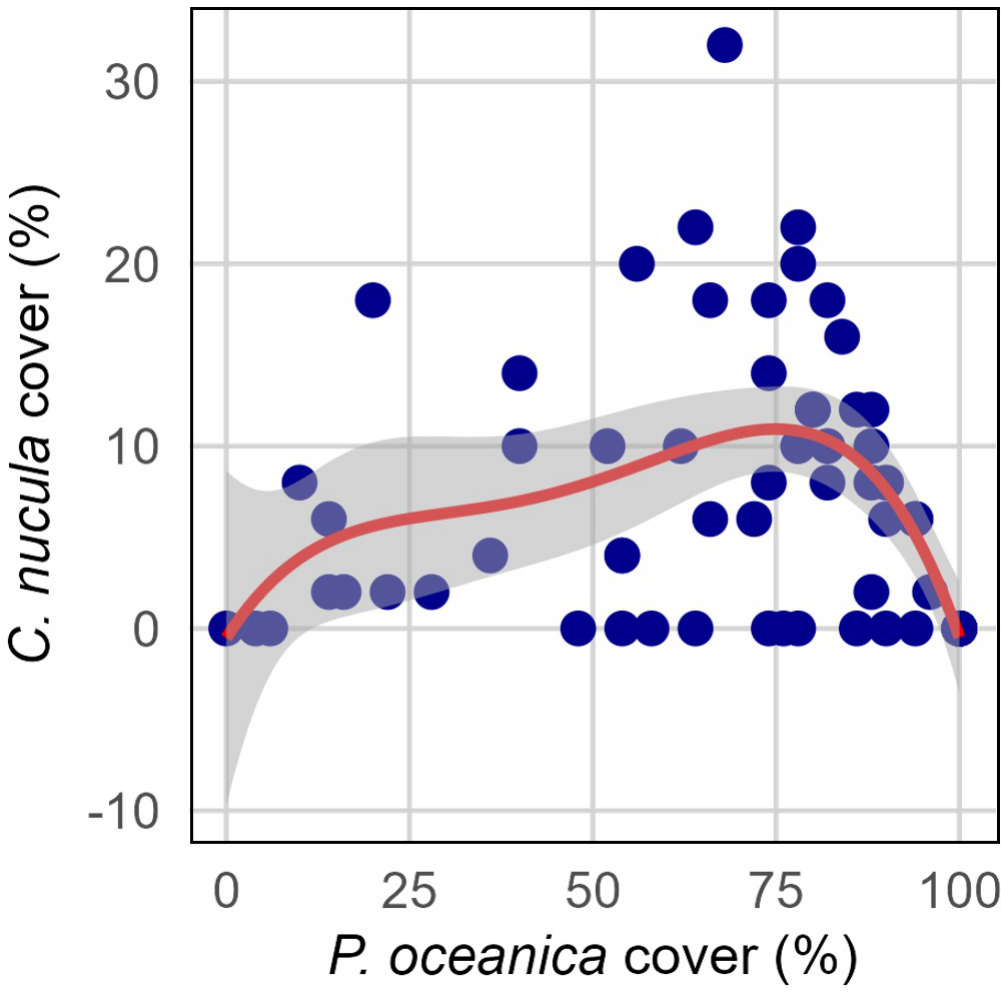
Relationship between *P. oceanica* cover (%) and *C. nucula* cover (%) based on field observations. Each point represents a discrete measurement, with *P. oceanica* cover plotted on the *x*-axis and *C. nucula* cover on the *y*-axis. A fourth-degree polynomial regression (red line) models the relationship, with shaded gray ribbons representing the 95% confidence interval.

Environmental variables at the sampling site were higher in autumn than in spring for ${\mathrm{NH}}_{4}^{+}$, nitrate+nitrite (${\mathrm{NO}}_{x}^{-}$), and DOC concentrations, as well as for seawater temperature (Table 1). The relationship between seagrass and sponge cover was non-linear (Fig. 2), and our model revealed a cubic curve (edf = 3.7, *p* = 6.55 × 10^−5^), with a peak in sponge cover (∼10% and up to 30%) at intermediate levels of seagrass cover (∼75%; Fig. 2). The asymmetry analysis supported this trend with significant q coefficients (Table S1).

### Primary production and respiration rates

**Figure 3:**
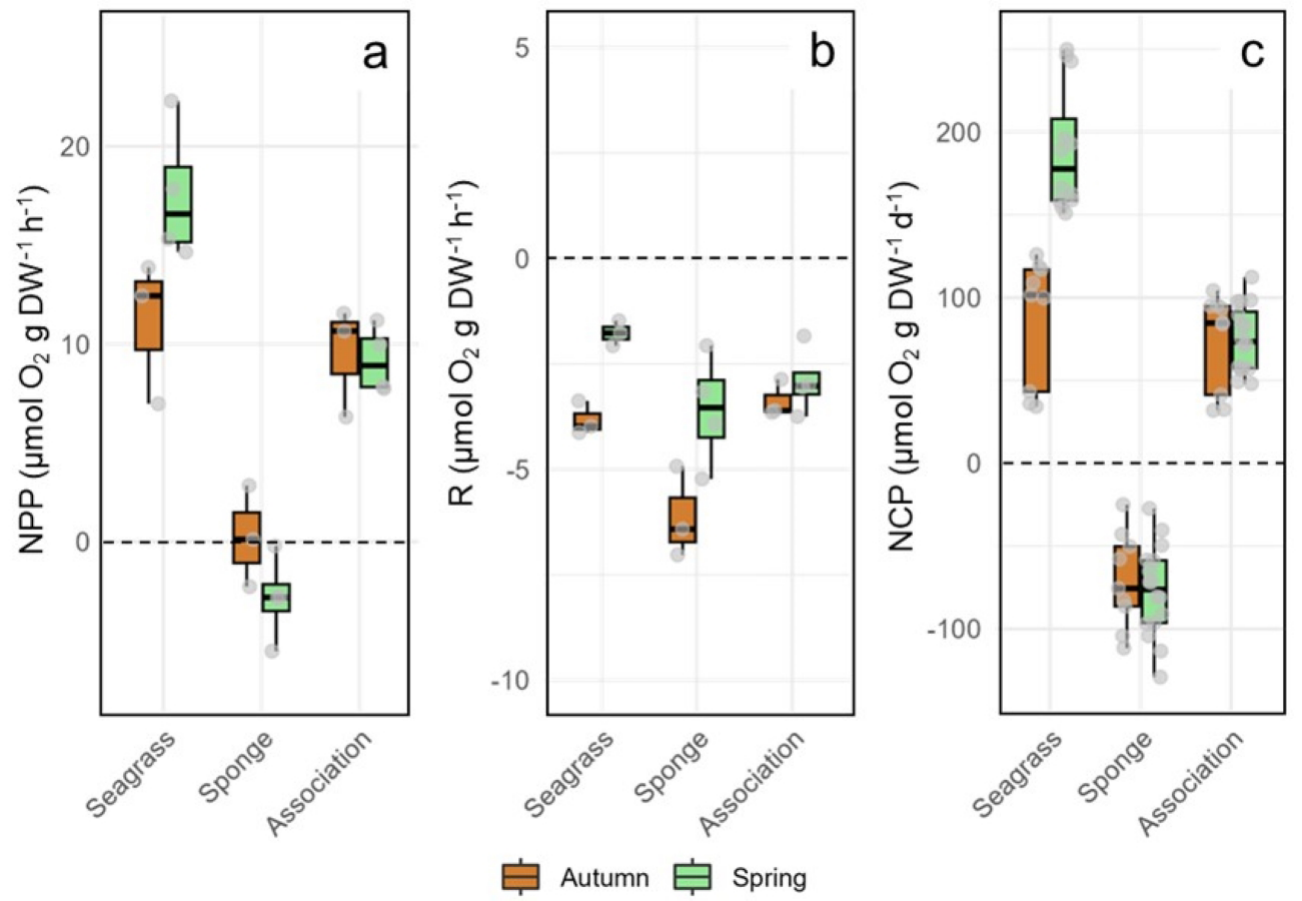
Seasonal variability in net primary production–NPP (A), dark respiration–R (B), and daily net community production–NCP (C) across the different *‘Community’* types (*P. oceanica*, *C. nucula*, and their association). Each panel displays the distribution of metabolic rates for two seasons (Autumn and Spring). Boxplots represent the interquartile range (IQR) with the median marked by a horizontal line. Whiskers extend to 1.5 × IQR, and outliers are shown as individual points (jittered for clarity). Positive values indicate oxygen production, while negative values reflect oxygen consumption. NPP and R are expressed in µmol O_2_ g DW^−1^ h ^−1^, while NCP is expressed in µmol O_2_ g DW^−1^ d^−1^.

A significant Community × Season interaction for hourly rates of net primary production (NPP, PERMANOVA: pseudo-F = 5.40, *p* = 0.022; Table S2) indicated that the seasonal pattern was community-specific. The significant interaction was driven primarily by a mean decrease of 37% in seagrass NPP from spring to autumn (17.5 ± 3.5 *vs* 11.1 ± 3.6 µmol O_2_ g DW^−1^ h^−1^, respectively; Fig. 3A), though this pairwise difference was borderline (Tukey HSD: *p* = 0.067; Table S3). Mean NPP rates in the sponge were near zero in autumn and negative in spring (0.2 ± 2.6 *vs* −2.8 ± 2.2 µmol O_2_ g DW^−1^ h^−1^, respectively; Fig. 3A), though this difference was not statistically significant (Tukey HSD: *p* = 0.136; Table S3), whereas NPP rates in the association showed no seasonal variation (pseudo-F = 5.40, *p* = 0.022; Table S3). Hourly respiration (R) rates differed significantly among Community types (pseudo-F = 9.20, *p* = 0.001) and between seasons (pseudo-F = 18.51, *p* = 0.001), with no significant Community × Season interaction (pseudo-F = 2.68, *p* = 0.108; Table S2), being highest (more negative) in the sponge, followed by the seagrass-sponge association, and lowest in the seagrass (Fig. 3B, Table S2). Again, a seasonal effect was pronounced in the seagrass, with R rates in autumn 2-fold higher (more negative) than in spring (−3.8 ± 0.4 *vs* −1.8 ± 0.3 µmol O_2_ g DW^−1^ h^−1^, respectively; Fig. 3B, Table S3), as well as in the sponge (−6.1 ± 1.1 *vs* −3.6 ± 1.3 µmol O_2_ g DW^−1^ h^−1^, respectively; Fig. 3B, Table S3). R rates in the association showed little seasonal variation (Fig. 3B). A significant Community × Season interaction for GPP (pseudo-F = 21.84, *p* = 0.001; Table S2) indicated community-specific seasonal dynamics, with higher GPP in the seagrass in spring compared to autumn (Tukey HSD: *p* = 0.008; Table S3), and no significant seasonal variation in the sponge or association. A significant Community × Season interaction for daily rates of net community production (NCP, pseudo-F = 21.72, *p* = 0.001; Table S2) indicated that the seasonal response was community-specific. NCP was 54% lower in autumn compared to spring in the seagrass (87.2 ± 38.0 *vs* 189.0 ± 37.7 µmol O_2_ g DW^−1^ day^−1^, respectively; Tukey HSD: *p* = 0.001, Fig. 3C, Table S3), while it remained stable across seasons in the sponge (−70.8 ± 28.8 *vs* −76.0 ± 28.7 µmol O_2_ g DW^−1^ day^−1^, respectively; Tukey HSD: *p* = 0.60, Fig. 3C, Table S3) and in the association (73.6 ± 29.5 *vs* 78.8 ± 19.6 µmol O_2_ g DW^−1^ day^−1^, respectively; Tukey HSD: *p* = 0.858, Fig. 3C, Table S3).

### Organic nutrient fluxes

**Figure 4:**
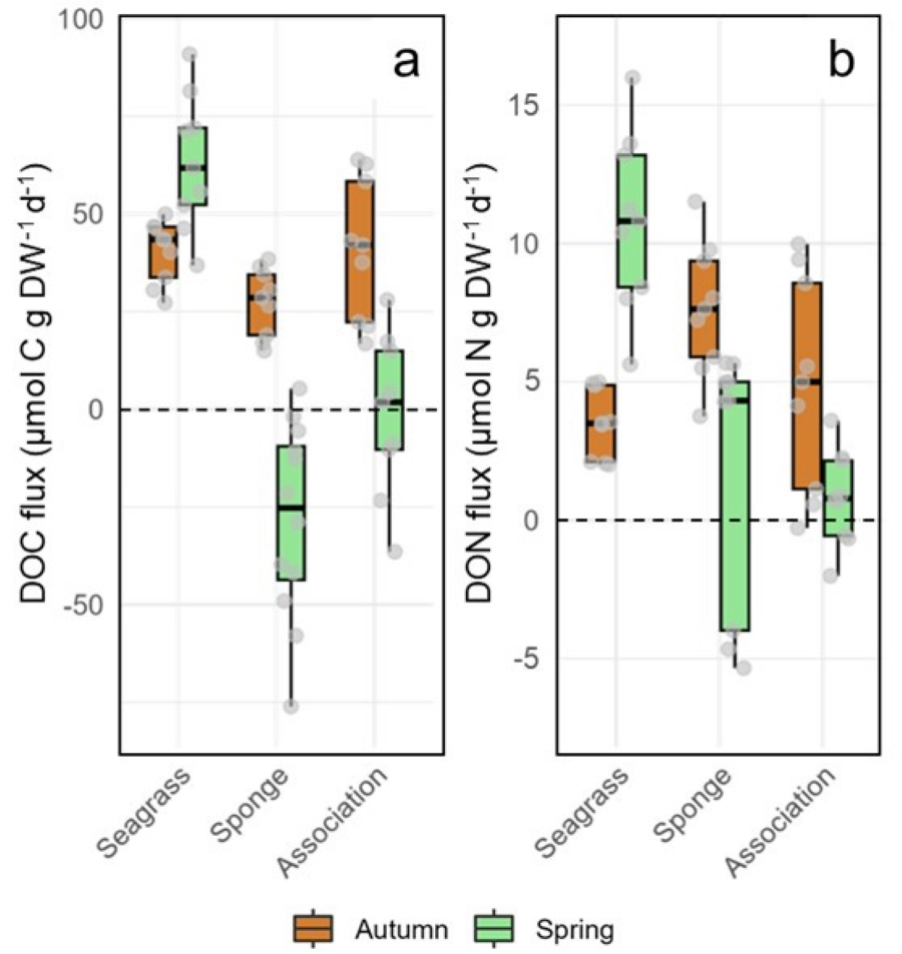
Seasonal variability in dissolved organic carbon–DOC (A) and dissolved organic nitrogen–DON (B) daily fluxes across the different *‘Community’* types (*P. oceanica*, *C. nucula* , and their association). Each panel shows the distribution of nutrient fluxes for two seasons (Autumn and Spring). Boxplots represent the interquartile range (IQR) with the median indicated by a horizontal line. Whiskers extend to 1.5 × IQR, with points outside this range plotted as individual data points (jittered for visibility). Negative values indicate nutrient uptake, and positive values indicate release. Flux rates are expressed in µmol g DW^−1^ d^−1^.

A significant Community × Season interaction for daily DOC fluxes (pseudo-F = 24.58, *p* = 0.001; Table S4) indicated that the pattern of DOC fluxes was community- and season-specific, showing the highest release rates in the seagrass treatment for both seasons and particularly in spring (autumn = 40.2 ± 7.9, spring = 63.1 ± 17.3 µmol C g DW^−1^ day^−1^, Fig. 4A, Table S5). In contrast, the sponge exhibited DOC release in autumn and a mean net uptake in spring (27.3 ± 8.6 µmol C g DW^−1^ day^−1^ *vs* -28.4 ± 24.9 µmol C g DW^−1^ day^−1^, respectively, Table S5), though the latter showed high variability that should be considered when interpreting the direction of this flux. The association also showed seasonality in DOC fluxes, with significant release in autumn (40.9 ± 18.2 µmol C g DW^−1^ day^−1^) and a near-zero, statistically indeterminate net flux in spring (−1.4 ± 20.5 µmol C g DW^−1^ day^−1^; Fig. 4A, Table S5), which precludes classification of the association as a clear DOC source or sink in this season. A significant Community × Season interaction for daily DON fluxes (pseudo-F = 16.48, *p* = 0.001; Table S4) indicated community-specific seasonal patterns. For the most part, DON fluxes showed release across communities and seasons (Fig. 4B, Table S4). In autumn, the highest DON release was in the sponge (7.6 ± 2.4 µmol N g DW^−1^ day^−1^), with intermediate values in the association and the lowest in seagrass (3.5 ± 1.2 µmol N g DW^−1^ day^−1^, Table S5). During spring, the seagrass exhibited the highest DON release (10.8 ± 3.2 µmol N g DW^−1^ day^−1^), with intermediate values in the sponge and the lowest in the association (0.8 ± 1.7 µmol N g DW^−1^day^−1^; Fig. 4B, Table S5). Hourly DOC fluxes were significantly affected by Community (pseudo-F = 7.78, *p* = 0.002), Season (pseudo-F = 11.06, *p* = 0.005), and Condition (pseudo-F = 11.58, *p* = 0.003; Table S4). Hourly DON fluxes showed no significant main effects of Community (*p* = 0.559) or Season (*p* = 0.155), but significant Community × Condition (pseudo-F = 11.21, *p* = 0.001) and Season × Condition interactions (pseudo-F = 41.12, *p* = 0.001; Table S4). Hourly DOC fluxes (Fig. S1, Tables S4, S5) were higher in the light than in the dark in the seagrass and association *Community* types, with highest values for the seagrass in the light in spring. The sponge showed release in autumn but uptake in spring, with no clear differences between dark and light. Hourly DON fluxes were higher in the dark than in the light in autumn for all three *Community* types (Fig. S1, Tables S4, S5), while they were higher in the light than in the dark in the seagrass and in the association in spring.

### Inorganic nutrient fluxes

**Figure 5:**
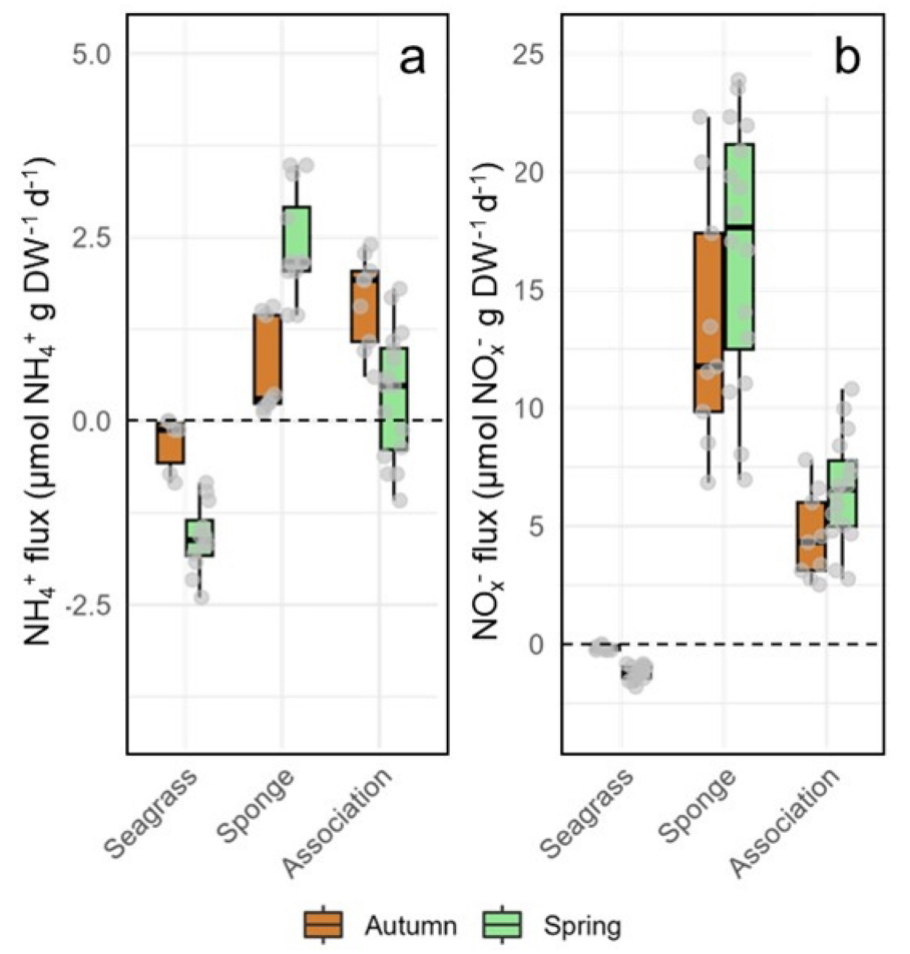
Seasonal variability in ammonium (A), and nitrate + nitrite (B) daily fluxes across the different *‘Community’* types (*P. oceanica*, *C. nucula*, and their association). Each panel shows the distribution of nutrient fluxes for two seasons (Autumn and Spring). Boxplots represent the interquartile range (IQR) with the median indicated by a horizontal line. Whiskers extend to 1.5 × IQR, with points outside this range plotted as individual data points (jittered for visibility). Negative values indicate nutrient uptake, and positive values indicate release. Flux rates are expressed in µmol g DW^−1^ d^−1^.

A significant Community × Season interaction for daily NH_4_^+^ fluxes (pseudo-F = 33.06, *p* = 0.001; Table S6) indicated community-specific seasonal patterns. ${\mathrm{NH}}_{4}^{+}$ fluxes (Fig. 5A) showed net uptake in the seagrass across *Seasons*, which was statistically significant in spring but near zero in autumn (autumn = −0.3 ± 0.4, spring = −1.6 ± 0.5 µmol ${\mathrm{NH}}_{4}^{+}$ g DW^−1^ day^−1^; Table S6, S7). Conversely, the sponge showed consistent ${\mathrm{NH}}_{4}^{+}$ release (autumn = 0.7 ± 0.6, spring = 2.4 ± 0.7 µmol ${\mathrm{NH}}_{4}^{+}$ g DW^−1^ day^−1^), while the association showed release in autumn (1.6 ± 0.6 µmol ${\mathrm{NH}}_{4}^{+}$ g DW^−1^ day^−1^) and a flux not significantly different from zero in spring (0.4 ± 0.9 µmol ${\mathrm{NH}}_{4}^{+}$ g DW^−1^ day^−1^; Tables S6, S7). Daily NO_*x*_^−^ fluxes differed significantly among Community types (pseudo-F = 121.04, *p* = 0.001) and between seasons (pseudo-F = 4.43, *p* = 0.048), with no significant interaction (pseudo-F = 2.35, *p* = 0.116; Table S6). The seagrass showed low but consistent ${\mathrm{NO}}_{\mathrm{x}}^{-}$ uptake (autumn = −0.2 ± 0.1, spring = −1.2 ± 0.3 µmol ${\mathrm{NO}}_{\mathrm{x}}^{-}$ g DW^−1^day^−1^; Fig. 5B), while the sponge displayed strong and consistent ${\mathrm{NO}}_{x}^{-}$ release (autumn = 13.6  ± 5.4, spring = 16.7 ± 5.5 µmol ${\mathrm{NO}}_{\mathrm{x}}^{-}$ g DW^−1^day^−1^), with the association showing intermediate but positive fluxes in both seasons (autumn = 4.6 ± 1.9, spring = 6.5 ± 2.3 µmol ${\mathrm{NO}}_{x}^{-}$ g DW^−1^day^−1^; Tables S6, S7). In autumn, both the seagrass and the sponge showed ${\mathrm{NH}}_{4}^{+}$ release in the light and ${\mathrm{NH}}_{4}^{+}$ uptake in the dark (Fig. S2, Tables S6, S7), while this pattern was not maintained in spring. Conversely, ${\mathrm{NO}}_{x}^{-}$ fluxes were highest in the sponge with higher release in the dark than in the daylight, across both autumn and spring (Fig. S2, Tables S6, S7). Daily PO_4_^3^^−^ fluxes differed significantly among Community types (pseudo-F = 37.66, *p* = 0.001) and between seasons (pseudo-F = 21.96, *p* = 0.001), with no significant interaction (pseudo-F = 1.59, *p* = 0.186; Table S6). PO_4_^3−^ fluxes were low (close to detection limit) and variable, with no clear difference between daylight and dark but mainly uptake in the seagrass and release in the sponge and the association (Fig. S2, Tables S6, S7).

### Stable isotope analysis

The *δ*^13^C values of the seagrass ranged from −13.7 ± 1.3‰  when associated with the sponge to −13.9 ± 0.8‰  when non-associated (Fig. S3), with no statistical differences between the two groups (pseudo-F = 1.49, *p* = 0.236; Table S8). Concurrently, we detected an increase in *δ*^15^N of ca. 1‰  in *P. oceanica* living associated with the sponge, from 4.3 ± 1.0‰  to 5.3 ± 0.7‰  (Sample × Association interaction: pseudo-F = 5.04, *p* = 0.005; Fig. S3, Tables S8, S9). The sponge *C. nucula* showed similar *δ*^13^C and *δ*^15^N values for non-associated sponges (−19.2 ± 0.5‰  and 6.6 ± 0.7‰) *vs* sponges associated with the seagrass (−19.0 ±0.5‰  and 6.7 ± 0.6‰, *n* = 20; Fig. S3). Plant epiphytes had *δ*^13^C values similar to those of the sponge (−18.8 ± 2.4‰  and −19.6 ± 2.2‰, *n* = 10, for associated *vs* non-associated plants, respectively) but showed an increase in their *δ*^15^N of ca. 1‰  when the plant was associated with the sponge (from 5.9 ± 1.0‰  to 7.3 ± 1.5‰, Fig. S3, Tables S8, S9). C:N ratios differed significantly among Sample types (pseudo-F = 112.31, *p* = 0.001) but not between Association types (pseudo-F = 0.46, *p* = 0.509; Table S8).

### Estimation of reciprocal nutrient contributions

**Figure 6:**
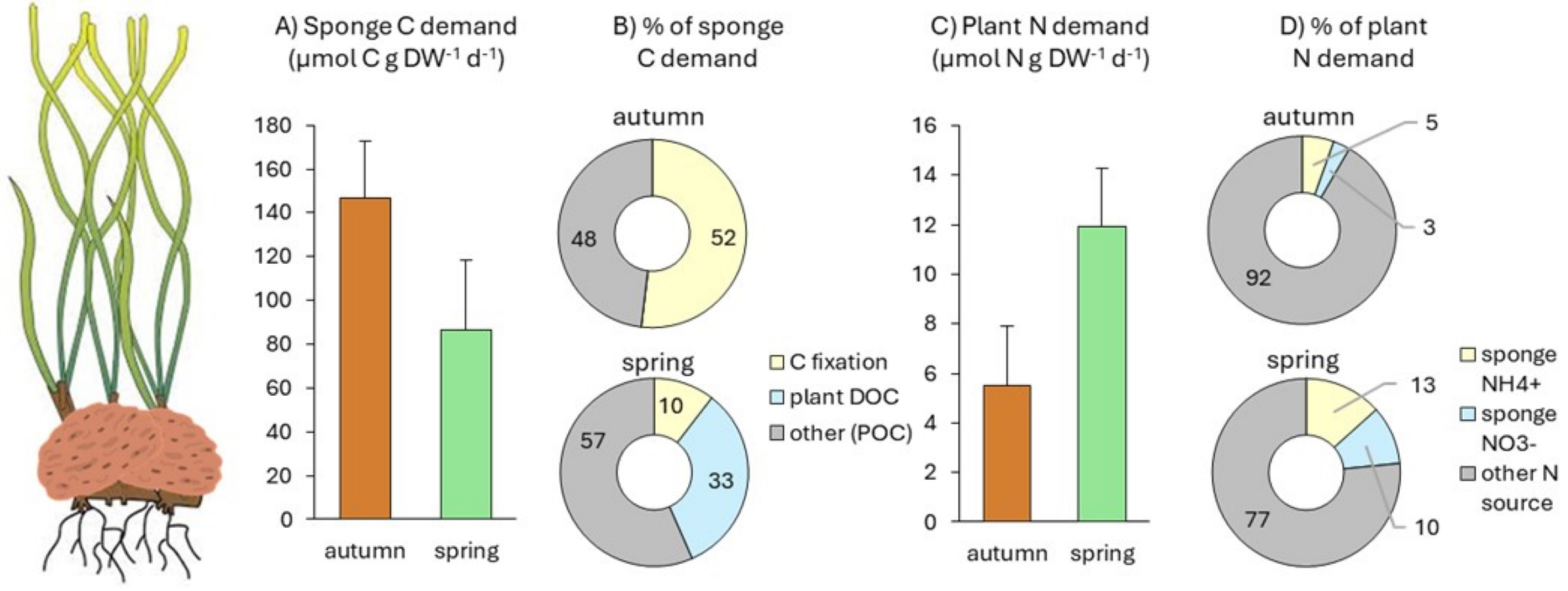
Photoautotrophic and heterotrophic nutrient recycling within the *P. oceanica*–*C. nucula* association. (A) Seasonal variation in the sponge daily respiratory carbon (C) demand (µmol C g DW^−1^ d^−1^), shown as mean ± SD. (B) Potential contribution (%) of heterotrophic dissolved organic carbon (DOC) and photoautotrophic C fixation to the sponge respiratory C demand, with the remaining fraction hypothesized to originate from particulate organic carbon (POC) *via* filter-feeding. (C) Seasonal variation in the plant daily nitrogen (N) demand estimated from net community production (NCP) and C:N ratios (µmol N g DW^−1^ d^−1^), shown as mean ± SD. (D) Potential contribution (%) of sponge ammonium (NH_4_) and nitrate+nitrite (NO_*x*_) release to the plant total daily N demand. See the supplementary methods for details. Organism graphics from the Integration and Application Network image library (https://ian.umces.edu/media-library/).

In autumn, photoautotrophic carbon fixation (GPP) accounted for approximately 52% of the sponge’s daily respiratory carbon demand (Fig. 6), with heterotrophic DOC uptake contributing a negligible fraction and the remainder attributed to POC *via* filter-feeding. In spring, the phototrophic contribution declined (∼10%) and DOC uptake increased (∼33%), consistent with the shift to net heterotrophy. For the seagrass, sponge-derived NH_4_^+^ and NO_*x*_^−^ release could conservatively account for approximately 10% and 13% of the plant’s daily nitrogen demand in spring, respectively, with the remainder derived from other nitrogen sources.

## Discussion

This study provides new insights into an association between the seagrass *P. oceanica* and the sponge *C. nucula*, revealing key aspects of their interaction. We found that the association displays non-linear spatial dependence, with higher sponge abundance linked to intermediate seagrass cover. Further, we found indirect evidence consistent with the sponge benefiting from DOC released by the seagrass in spring—as indicated by concurrent seagrass DOC release and sponge DOC uptake, and a near-zero net flux in the association—while the sponge contributed substantial inorganic nitrogen, which may support seagrass productivity. These findings support the hypothesis of a facultative mutualism between *P. oceanica* and *C. nucula*, advance our understanding of the ecological dynamics within *P. oceanica* meadows, and highlight the importance of sponges in maintaining meadow stability and nutrient cycling.

### The sponge-seagrass association shows spatial dependence

We found evidence that the association between *C. nucula* and *P. oceanica* displays non-linear spatial dependence, with the maximum sponge cover occurring at intermediate seagrass cover (∼75%) and areas of both low and high seagrass cover corresponding to minimal sponge presence. We acknowledge that our sampling approach may underestimate *C. nucula* cover in areas of dense seagrass, as the sponge grows primarily at the base of shoots and around rhizomes and may be partially obscured from above. Cover estimates based on video transects should therefore be considered conservative, particularly at high seagrass densities. However, a detection bias alone would predict a monotonic negative relationship between seagrass and sponge cover, and cannot account for the initial increase in sponge abundance at low-to-intermediate seagrass densities that drives the non-linear GAM fit. Future surveys should combine video transects with diver-based quadrat estimates to validate cover relationships at high canopy densities. The detected non-linear spatial dependence suggests that at intermediate levels of seagrass cover, there is a favorable balance of available substrate and resource availability for both organisms at this site. These results align with Archer, Stoner & Layman (2015), who reported that intermediate seagrass cover offers sufficient substrate for sponge colonization without significantly reducing water flow or light, which could otherwise impair sponge nutrition and/or seagrass photosynthesis. Notably, Archer, Stoner & Layman (2015) also documented a context-dependent, non-linear spatial relationship in the *Halichondria melanadocia*–*Thalassia testudinum* association, broadly mirroring the pattern we describe here for *C. nucula* and *P. oceanica*. Furthermore, Archer, Hensel & Layman (2018) demonstrated that ambient nutrient availability modulates the outcome of sponge–seagrass interactions, with sponge-facilitated nutrient enrichment being most beneficial under oligotrophic conditions. Our study site in the nutrient-poor Mediterranean is consistent with this context, suggesting that the nitrogen-recycling role of *C. nucula* may be particularly significant here. This is relevant because *C. nucula*, similarly to its congeneric species from the Caribbean and Australia, is a photophilic HMA sponge with a rich microbiome dominated by autotrophic cyanobacteria, which contribute to its energy production (Hudspith et al., 2022; Mazzella et al., 2024; Usher et al., 2004). However, a previous report found that photoautotrophy could account for only a small fraction of the total daily carbon uptake in the Caribbean congeneric *C. caribensis* (ca. 7%), while DOC uptake contributed the most to the sponge diet (ca. 92%, Hudspith et al., 2022). At our study site, this may provide a competitive advantage for *C. nucula* to associate with a large primary producer, such as *P. oceanica*, which is known to release large amounts of DOM (Barrón & Duarte, 2009). The asymmetric spatial dependence of the sponge and seagrass indicates neutrality for *P. oceanica* toward the presence of the sponge. However, this neutrality could be the result of a balance between positive and negative effects, rather than the absence of interaction, as Mathis & Bronstein (2020) suggested. In particular, the seagrass may compete with the sponge for space, while at the same time may benefit from its efficient nutrient recycling capacity.

### Association with the sponge stabilizes meadow productivity

NPP measurements show that the sponge was near zero metabolic balance in autumn but shifted to net heterotrophy in spring. In contrast, the seagrass and the seagrass-sponge association remained autotrophic throughout both seasons. Our respirometry results are consistent with rates and seasonal dynamics described in previous studies (Berlinghof et al., 2022; Koopmans et al., 2020; Olivé et al., 2016), providing confidence that the data collected on these occasions are representative of broader seasonal processes, and confirming a shift for the plant from a highly productive growth phase in spring to a senescent phase in autumn. Sponges where symbiont photosynthesis exceeds holobiont respiration are termed “net phototrophic” and are estimated to have >50% of their daily respiratory needs met by their photosynthetic partners (Wilkinson & Trott, 1985). Although microbial diversity in *C. nucula* was not assessed in this study, our rates are to be attributed to cyanobacterial symbiont photosynthesis (Usher et al., 2004), which contributed significantly to sponge nutrition, providing approximately 52% of daily respiratory carbon demand in autumn while only a minor fraction in spring. This underscores the importance of mixotrophy in this sponge, which, similar to what has been reported for a congeneric species in the Caribbean, may rely heavily on DOM uptake (Hudspith et al., 2022). The counter-intuitive pattern—higher photoautotrophic contribution in autumn despite lower seasonal irradiance—is consistent with the nutritional flexibility documented in *S. spongiarum*-hosting sponges, where heterotrophic substrate availability, rather than light alone, determines the net metabolic balance (Erwin & Thacker, 2008). In autumn, lower DOC release by *P. oceanica* may reduce the availability of labile plant-derived organic carbon to the sponge, thereby reducing its dependence on heterotrophic carbon uptake, while the photosynthetic contribution of its cyanobacterial symbionts (*Ca. Synechococcus spongiarum*) remains relatively stable, resulting in a near-balanced metabolic state. Shading experiments in closely related species have demonstrated that both symbiont photosynthetic activity (measured as chlorophyll a) and host growth respond dynamically to light availability (Curdt, Schupp & Rohde, 2022; Erwin & Thacker, 2008), suggesting that the higher phototrophic contribution in autumn likely reflects increased per-cell photosynthetic activity in response to reduced plant DOC availability, rather than an increase in symbiont abundance. This interpretation is consistent with the compositional stability of the *Ca. Synechococcus spongiarum* core microbiome across seasons documented in other Mediterranean sponges (Erwin et al., 2012). *P. oceanica* showed strong seasonality in productivity (NPP and NCP), but this variation was significantly less pronounced when *C. nucula* was present, indicating a buffering effect by the sponge due to its increased autotrophy in autumn. It is known that biodiversity can enhance productivity, resource use, and stability of seagrass ecosystems (Duffy, 2006). Similarly to land plants, where species interactions that present asynchrony in species fluctuations result in niche partitioning or facilitation and increase both productivity and temporal stability (Isbell, Polley & Wilsey, 2009), meadows colonized by *C. nucula* may exhibit lower primary production relative to non-colonized meadows during productive seasons, but increased sponge activity during the plant senescence season may buffer the ecosystem against nutrient limitations, favoring nutrient recycling and promoting long-term stability.

### Nutrient fluxes between seagrass and sponge underpin the association

*P. oceanica* contributed significant amounts of DOM (as DOC and DON) to its surrounding environment, particularly in spring, concomitant with the highest plant NPP rates. *P. oceanica* is known to enhance DOC fluxes relative to adjacent unvegetated sediments, as these plants produce nonstructural carbohydrates in excess (Barrón & Duarte, 2009; Sogin et al., 2022). In particular, we estimate that the plant released approximately 46% and 33% of its NCP as DOC in autumn and spring, respectively. This is lower than the 71% estimate by Barrón & Duarte (2009), for a *P. oceanica* community in Mallorca Island (Spain), although their estimate also included contributions from allochthonous inputs.

DON was for the most part released by all community types across both seasons, albeit with high variability. The pattern mirrored that of DOC fluxes, with the highest DON release observed in seagrass during spring, coinciding with its growth season, and a tendency for DON uptake by the sponge and the association also in spring. DON fluxes in benthic organisms such as seagrasses and sponges have been rarely documented. The rates measured here are lower but comparable to those reported by Liu et al. (2018) for the tropical seagrasses *Thalassia hemprichii* and *Enhalus acoroides*, attributed to the leaching of nonstructural carbohydrates and other labile organic matter from seagrass leaves. In addition to seagrass leaching, epiphytes and sponges may also contribute DON to the surrounding environment. These sources of DON may have supported sponge heterotrophy in spring, as well as microbial processes such as ammonification (Pfister et al., 2023) and nitrification (Berlinghof et al., 2024), thereby facilitating nitrogen cycling within the studied system.

Concurrently, we detected net DOC uptake by *C. nucula* during spring, under both light and dark conditions, while the sponge released DOC in autumn. DOC uptake/release aligns with the sponge’s mixotrophic condition, shifting between autotrophy-dominated in autumn (DOC release) and heterotrophy-dominated (DOC uptake) in spring, as indicated by our measurements of NPP. If we assume linear DOC removal by the sponge in response to rising DOC concentrations in the environment (Ribes et al., 2023) and similar DOC release rates by the plant when associated with the sponge compared to when it is not associated, we can estimate that the DOC released by the seagrass in spring may have covered approximately 33% of the sponge’s respiratory carbon demand, a decrease from 92% in the congeneric low-light dwelling *C. caribensis* forma *hermatypica* (Hudspith et al., 2022). Given the difference in depth niches between the two species and the shallow depth at our study site, it is reasonable to expect that *C. nucula* relies more on photoautotrophy compared to its congeneric species from the Caribbean. This reliance on photoautotrophy was estimated to cover approximately 52% of its respiratory carbon needs in autumn and around 10% in spring. We note, however, that our incubation design does not allow direct confirmation of the identity of the carbon source assimilated by the sponge. The co-occurrence of seagrass DOC release and sponge DOC uptake in spring, together with the near-zero net DOC flux of the association, is consistent with—but does not conclusively demonstrate—a direct trophic link between the two organisms. Stable isotope labelling or tracer experiments would be required to confirm this.

While the co-occurrence of seagrass DOC release and sponge DOC uptake in spring is consistent with a carbon subsidy to the sponge, the sponge excreted significant amounts of [NH_4_^+^/NO_*x*_^−^], potentially benefiting plant growth. Indeed, we measured substantial uptake of both ${\mathrm{NH}}_{4}^{+}$ and ${\mathrm{NO}}_{3}^{-}$ by the seagrass, particularly during spring, which coincides with its peak productivity season. HMA sponges are particularly known for contributing dissolved inorganic nutrients to their surroundings, primarily in the forms of ${\mathrm{NH}}_{4}^{+}$, ${\mathrm{NO}}_{3}^{-}$, and ${\mathrm{PO}}_{4}^{3-}$(Maldonado, Ribes & Van Duyl, 2012). Notably, *C. nucula* exhibited substantial NO_*x*_^−^ fluxes in both autumn and spring. These fluxes are likely the result of microbial nitrification within the sponge’s body. Numerous studies have demonstrated that sponges often associate with ammonium-oxidizing and nitrite-oxidizing microorganisms (Jiménez & Ribes, 2007; Schläppy et al., 2010; Southwell, Popp & Martens, 2008), and *C. nucula* at our study site is probably no exception. Specifically, ${\mathrm{NO}}_{2}^{-}$ and ${\mathrm{NO}}_{3}^{-}$ production rates in *C. nucula* aligned closely with those reported in previous studies from the Caribbean (Corredor et al., 1988; Diaz & Ward, 1997), and showed that 98% of ${\mathrm{NO}}_{x}^{-}$ was released as ${\mathrm{NO}}_{3}^{-}$. This pattern indicates the coexistence of ammonia-oxidation and nitrite-oxidation within the sponge holobiont. Further studies are needed to quantify these processes using stable isotope labeling. However, if we conservatively assume that the plant’s uptake remains constant when in association with the sponge compared to when it is not, we can estimate that the sponge contributed approximately 10% to the plant N demand in spring through ${\mathrm{NO}}_{x}^{-}$ uptake and about 13% through ${\mathrm{NH}}_{4}^{+}$ uptake. This mechanism—inorganic nitrogen release by the sponge supporting seagrass nutrition—mirrors what Archer, Stoner & Layman (2015), Archer, Hensel & Layman (2018) documented for the *H. melanadocia*–*T. testudinum* system in the Caribbean, where sponge-derived NH_4_^+^ and PO_4_^3^^−^ enhanced seagrass tissue nutrient content and growth.

Our analysis of stable isotope data reveals that both the plant and its epiphytes exhibited higher *δ*^1^^5^N values when associated with the sponge compared to when they were found alone. Significant isotopic fractionation occurs during microbial nitrification, resulting in the product ${\mathrm{NO}}_{3}^{-}$ being depleted in *δ*^1^^5^N while the residual ${\mathrm{NH}}_{4}^{+}$ becomes enriched in *δ*^1^^5^N (Casciotti & Buchwald, 2012). In fact, the isotopic composition of ${\mathrm{NO}}_{3}^{-}$ expelled from sponges *in situ* has lower *δ*^1^^5^N values than ${\mathrm{NO}}_{3}^{-}$ from the ambient water column due to nitrification (Southwell, Popp & Martens, 2008). Therefore, in our study, the increase in *δ*^1^^5^N values in both the plant and its epiphytes when associated with the sponge may have resulted from the preferential incorporation of *δ*^1^^5^N-enriched residual ${\mathrm{NH}}_{4}^{+}$ excreted by the sponge, which becomes enriched in ^1^^5^N during microbial nitrification. Seagrasses often exhibit preferential uptake of ${\mathrm{NH}}_{4}^{+}$, with ${\mathrm{NO}}_{3}^{-}$ uptake rates representing only a small fraction of total nitrogen uptake (Alexandre, Georgiou & Santos, 2014; Alexandre et al., 2011), also because ${\mathrm{NO}}_{3}^{-}$ incorporation implies active transport with an associated energetic cost (Touchette & Burkholder, 2000). However, this pattern may not prevent the *P. oceanica* holobiont from benefiting also from the released ${\mathrm{NO}}_{3}^{-}$. This seagrass species exhibits a complex nitrogen budget that involves both uptake and recycling processes (Berlinghof et al., 2024; Pfister et al., 2023), which may enable *P. oceanica* to utilize ${\mathrm{NO}}_{3}^{-}$ particularly in spring (Lepoint et al., 2002). This capability may allow the plant to meet its nitrogen requirements even in the nutrient-poor conditions of the Mediterranean Sea. In contrast, no corresponding shift in *δ*^1^^3^C was detected in *C. nucula* when associated with the seagrass. While *P. oceanica* tissue is isotopically ^1^^3^C-enriched relative to ambient marine particulate organic matter in the Mediterranean (Vizzini et al., 2002), any seagrass-derived DOC contribution to the sponge carbon budget would be diluted by other carbon sources—including photosymbiont-fixed carbon and particulate organic matter *via* filter feeding—rendering it insufficient to produce a detectable shift in bulk sponge *δ*^1^^3^C at natural isotopic abundances. Confirmation of a direct carbon transfer from seagrass to sponge would therefore require tracer experiments using ^1^^3^C-enriched substrates.

## Conclusions

Our study highlights the ecological significance of the association between the seagrass *P. oceanica* and the sponge *C. nucula*, offering new evidence of spatial dependence and nutrient exchange between these organisms. The results suggest that intermediate seagrass cover promotes sponge colonization while ensuring favorable conditions for both organisms. The association is characterized by a dynamic seasonal balance between autotrophy and heterotrophy, with the sponge showing net DOC uptake concurrent with seagrass DOC release, consistent with a trophic link, and contributing inorganic nitrogen that likely enhances seagrass productivity in spring. This facultative mutualism stabilizes meadow productivity by buffering seasonal fluctuations and underscores the critical role of sponges in nutrient recycling within seagrass ecosystems. Future research should focus on tracing nutrient flows between the two species using stable isotope labeling, quantifying the contribution of microbial nitrification to the sponge’s nitrogen output, as well as assessing the stability of the association under environmental stressors. Repeated sampling within each season, and extension to summer and winter time points, would further allow assessment of intra-seasonal variability, capture the full annual cycle of the association, and help disentangle short-term environmental fluctuations from the broader seasonal patterns documented here. By deepening our understanding of these interactions, we can better predict how such associations may respond to global changes and contribute to the stability of seagrass ecosystems.

## Supplemental Information

10.7717/peerj.21392/supp-1 Supplemental Information 1 Supplemental figures and tables

## Additional Information and Declarations

### Data Availability

The following information was supplied regarding data availability: The data is available at Zenodo: Cardini, U., Montilla, L. M., Zapata-Hernández, G., Berlinghof, J., Guarcini, E., Furia, M., Margiotta, F., Meador, T., Wild, C., Fraschetti, S., & Olivé, I. (2024). Reciprocal nutritional benefits in a sponge-seagrass association [Data set]. Zenodo. https://doi.org/10.5281/zenodo.14005724.
