# Supplementary material for: Reciprocal nutritional benefits in a Mediterranean seagrass-sponge association
Source: PeerJ. 2026 Jul 17;14:e21392. doi: 10.7717/peerj.21392 (PMC13383961; doi:10.7717/peerj.21392)
Supplement: Supplemental Information 1 [file peerj-14-21392-s001.docx]

**Supplementary materials for:**

**Reciprocal nutritional benefits in a sponge-seagrass association**

Cardini U., Montilla L.M., Zapata-Hernández G., Berlinghof J., Guarcini E., Furia M., Margiotta F., Meador T., Wild C., Fraschetti S., Olivé I.

Correspondence to: [ulisse.cardini@szn.it](mailto:ulisse.cardini@szn.it)

**
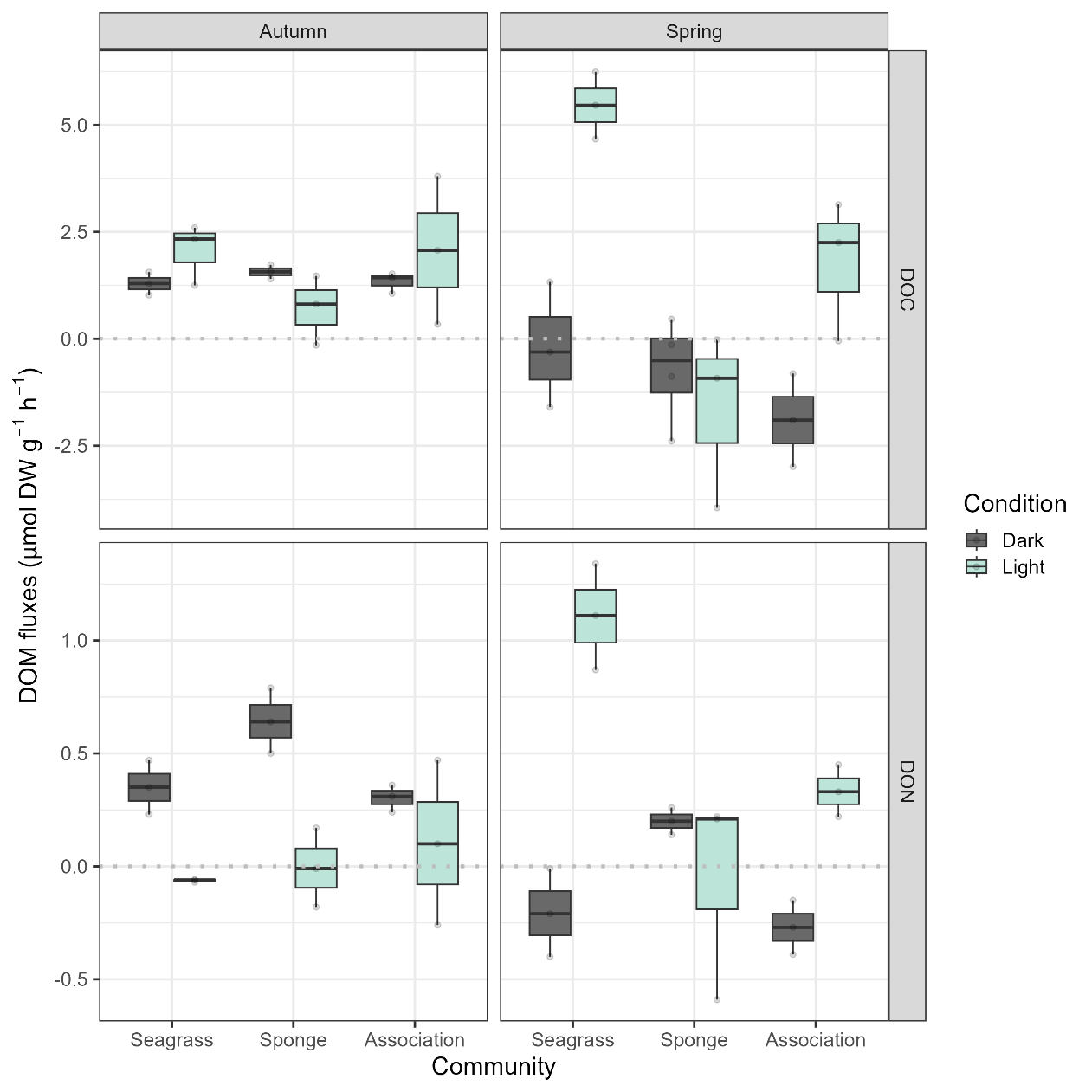
**

**Fig. S1.** Dissolved organic matter (DOM) fluxes expressed as µmol g DW⁻¹ h⁻¹ for dissolved organic carbon (DOC, top panels) and dissolved organic nitrogen (DON, bottom panels) across three communities—*Posidonia oceanica* (Seagrass), *Chondrilla nucula* (Sponge), and their Association. Fluxes are shown separately for two seasons: Autumn (left) and Spring (right). Each boxplot displays the fluxes under dark (gray) and light (teal) conditions. Positive values represent net release, while negative values indicate uptake. The horizontal dashed line marks the zero-flux threshold. Whiskers denote variability across replicates, and the central line in each box indicates the median flux.

**
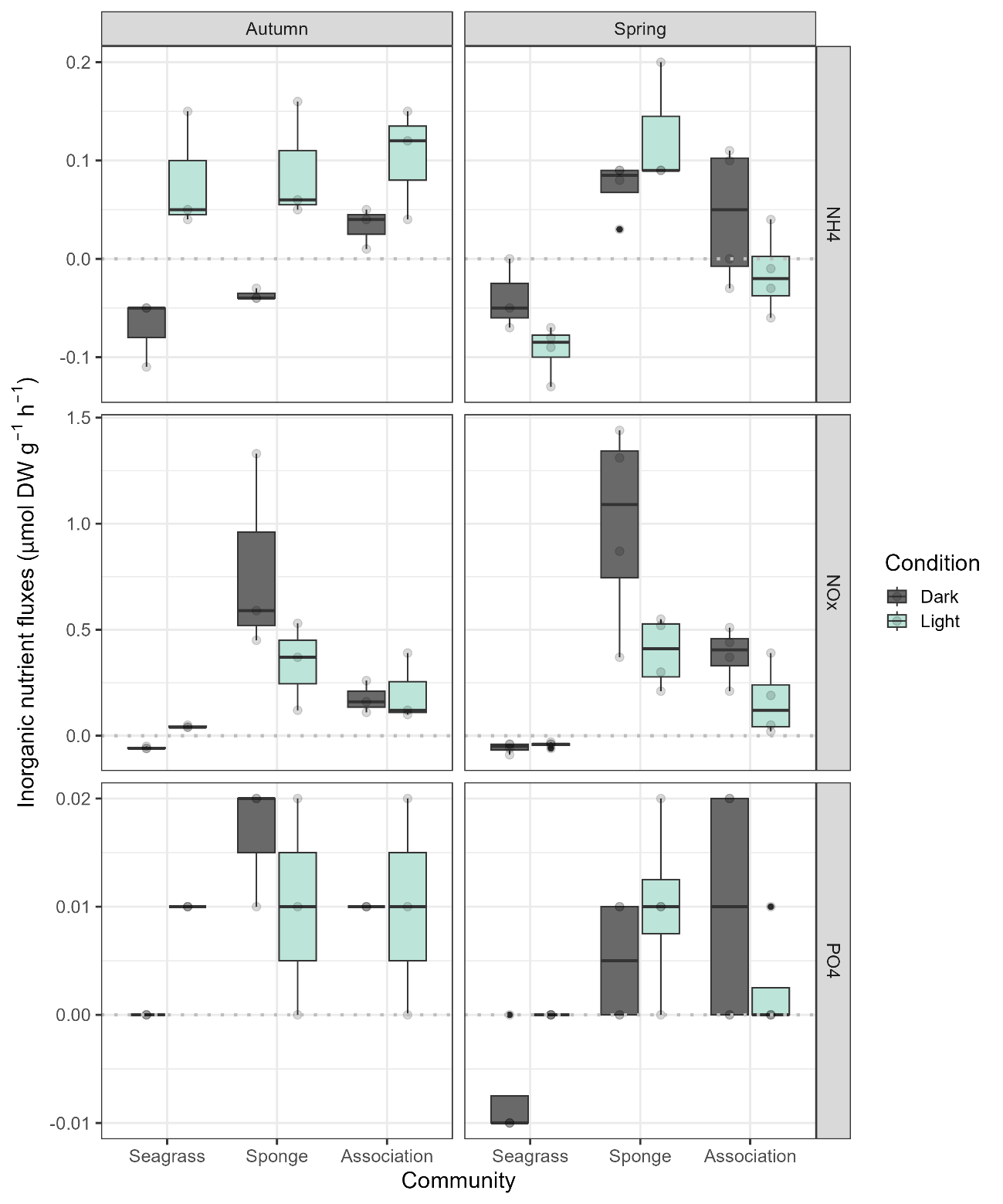
**

**Fig. S2.** Inorganic nutrient fluxes expressed as µmol g DW⁻¹ h⁻¹ for ammonium (NH₄⁺, top panels), nitrate+nitrite (NOₓ^-^, middle panels), and phosphate (PO₄³⁻, bottom panels) across three communities—*Posidonia oceanica* (Seagrass), *Chondrilla nucula* (Sponge), and their Association. Fluxes are presented separately for Autumn (left) and Spring (right) seasons under dark (gray) and light (teal) conditions. Positive values represent net release, while negative values indicate uptake. The horizontal dashed line marks the zero-flux threshold. Whiskers display variability across replicates, with the central line in each box representing the median flux.

**
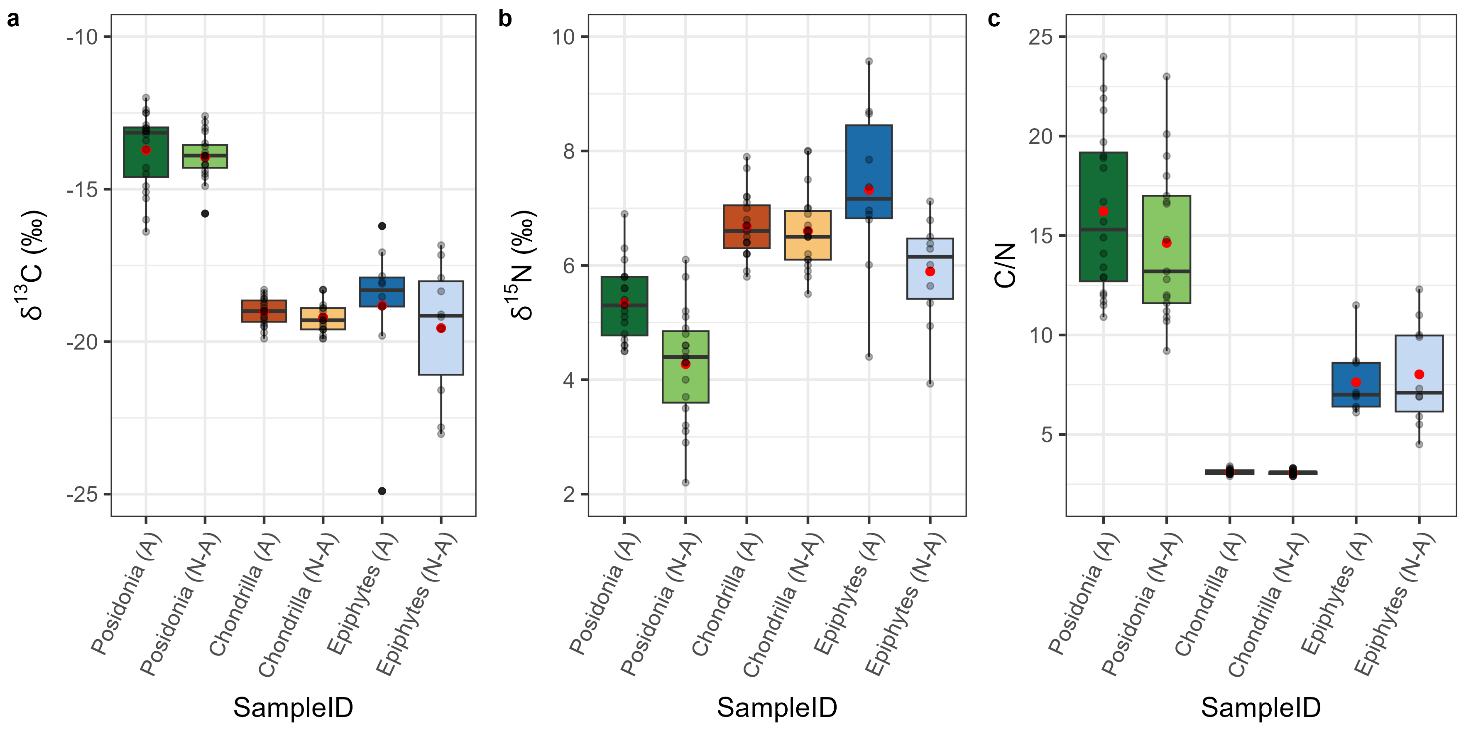
**

**Fig. S3.** Stable isotope composition and C/N ratios across communities in the association (A) and non-association (N-A) states. (a) δ¹³C values (‰), (b) δ¹⁵N values (‰), and (c) C/N ratios for *Posidonia oceanica*, *Chondrilla nucula*, and seagrass epiphytes. Each boxplot shows the distribution of values, with individual data points represented by black dots and the mean indicated by a red dot. Whiskers display the range of variability across replicates.

**SUPPLEMENTARY TABLES**

**Table S1.** Coefficients of asymmetric dependency between the benthic cover of the seagrass *Posidonia oceanica* and the sponge *Chondrilla nucula*. The coefficient q(X,Y) indicates the strength of the dependency of organism Y on organism X. The asymmetry coefficient quantifies the imbalance between these dependencies, with a non-significant value suggesting that the interaction does not exhibit a strong directional asymmetry.

| Coefficients | q | p-value |
| --- | --- | --- |
| q(*C. nucula*, *P. oceanica*) | 0.249 | **0.021** |
| q(*P. oceanica, C. nucula*) | 0.381 | **0.001** |
| asymmetry | -0.132 | 0.106 |

**Table S2.** Permutation-based analysis of variance (PERMANOVA) for net primary production (NPP, µmol O₂ g DW⁻¹ h⁻¹), respiration (R, µmol O₂ g DW⁻¹ h⁻¹), gross primary production (GPP, µmol O₂ g DW⁻¹ h⁻¹) and daily net community production (NCP, µmol O₂ g DW⁻¹ d⁻¹) across different *Community* types, *Seasons*, and their interaction. The table provides the degrees of freedom (Df), sum of squares (SS), proportion of variance explained (R²), pseudo-F statistics, and associated p-values (P(>F)) for each source of variation. Bold p-values (p < 0.05) indicate which factors contribute to differences in the measured variables.

| **Variable** | **Source of variation** | **Df** | **SS** | **R^2^** | **Pseudo-F** | **P (>F)** |
| --- | --- | --- | --- | --- | --- | --- |
| **Net primary production**  **(NPP)** | Community | 2 | 963.6 | 0.83 | 63.48 | **0.001** |
|  | Season | 1 | 5.3 | 0.00 | 0.70 | 0.389 |
|  | Community:Season | 2 | 81.9 | 0.07 | 5.40 | **0.022** |
|  | Residual | 15 | 113.9 | 0.10 |  |  |
|  | Total | 20 | 1164.6 | 1.00 |  |  |
| **Respiration (R)** | Community | 2 | 13.6 | 0.33 | 9.20 | **0.001** |
|  | Season | 1 | 13.7 | 0.33 | 18.51 | **0.001** |
|  | Community:Season | 2 | 4.0 | 0.10 | 2.68 | 0.108 |
|  | Residual | 14 | 10.4 | 0.25 |  |  |
|  | Total | 19 | 41.7 | 1.00 |  |  |
| **Gross primary production (GPP)** | Community | 2 | 2588.8 | 0.79 | 208.26 | **0.001** |
|  | Season | 1 | 12.7 | 0.00 | 2.05 | 0.159 |
|  | Community:Season | 2 | 271.5 | 0.08 | 21.84 | **0.001** |
|  | Residual | 65 | 404.0 | 0.12 |  |  |
|  | Total | 70 | 3277.0 | 1.00 |  |  |
| **Net community production (NCP)** | Community | 2 | 592852 | 0.84 | 332.33 | **0.001** |
|  | Season | 1 | 14804 | 0.02 | 16.60 | **0.003** |
|  | Community:Season | 2 | 38754 | 0.06 | 21.72 | **0.001** |
|  | Residual | 65 | 57978 | 0.08 |  |  |
|  | Total | 70 | 704388 | 1.00 |  |  |

**Table S3**. Adjusted p-values from multilevel pairwise comparisons of net primary production (NPP), gross primary production (GPP) and net community production (NCP) between *Community* and *Season*. The comparisons are performed using Tukey’s honest significant difference (HSD) test. Bold p-values indicate combinations that differ significantly (p < 0.05).

| **NPP** | Seagrass (Autumn) | Seagrass  (Spring) | Sponge (Autumn) | Sponge  (Spring) | Association (Autumn) | Association  (Spring) |
| --- | --- | --- | --- | --- | --- | --- |
| Seagrass (Autumn) | x |  |  |  |  |  |
| Seagrass (Spring) | 0.067 | x |  |  |  |  |
| Sponge (Autumn) | 0.100 | **0.034** | x |  |  |  |
| Sponge  (Spring) | **0.029** | **0.032** | 0.136 | x |  |  |
| Association (Autumn) | 0.500 | **0.030** | 0.100 | **0.024** | x |  |
| Association (Spring) | 0.356 | **0.025** | **0.031** | **0.030** | 0.855 | x |
| **GPP** | Seagrass (Autumn) | Seagrass  (Spring) | Sponge (Autumn) | Sponge  (Spring) | Association (Autumn) | Association  (Spring) |
| Seagrass (Autumn) | x |  |  |  |  |  |
| Seagrass (Spring) | **0.008** | x |  |  |  |  |
| Sponge (Autumn) | **0.001** | **0.001** | x |  |  |  |
| Sponge  (Spring) | **0.001** | **0.001** | **0.001** | x |  |  |
| Association (Autumn) | 0.150 | **0.001** | **0.002** | **0.001** | x |  |
| Association (Spring) | **0.008** | **0.001** | **0.001** | **0.001** | 0.375 | x |
| **NCP** | Seagrass (Autumn) | Seagrass  (Spring) | Sponge (Autumn) | Sponge  (Spring) | Association (Autumn) | Association  (Spring) |
| Seagrass (Autumn) | x |  |  |  |  |  |
| Seagrass (Spring) | **0.001** | x |  |  |  |  |
| Sponge (Autumn) | **0.001** | **0.001** | x |  |  |  |
| Sponge  (Spring) | **0.001** | **0.001** | 0.602 | x |  |  |
| Association (Autumn) | 0.419 | **0.001** | **0.001** | **0.001** | x |  |
| Association (Spring) | 0.320 | **0.001** | **0.001** | **0.001** | 0.858 | x |

**Table S4.** PERMANOVA for hourly (µmol g DW⁻¹ h⁻¹) and daily (µmol g DW⁻¹ d⁻¹) DOC and DON fluxes across different *Community* types, *Seasons*, *Condition* (light vs dark, when present) and their interaction terms. The table provides the degrees of freedom (Df), sum of squares (SS), proportion of variance explained (R²), pseudo-F statistics, and associated p-values (P(>F)) for each source of variation. Bold p-values (p < 0.05) indicate which factors contribute to differences in the measured variables.

| **Variable** | **Source of variation** | **Df** | **SS** | **R^2^** | **Pseudo-F** | **P (>F)** |
| --- | --- | --- | --- | --- | --- | --- |
| **Hourly DOC fluxes** | Community | 2 | 22.39 | 0.15 | 7.78 | **0.002** |
|  | Season | 1 | 15.92 | 0.11 | 11.06 | **0.005** |
|  | Condition | 1 | 16.66 | 0.11 | 11.58 | **0.003** |
|  | Community:Season | 2 | 14.28 | 0.10 | 4.96 | **0.012** |
|  | Community:Condition | 2 | 21.24 | 0.15 | 7.38 | **0.004** |
|  | Season:Condition | 1 | 15.18 | 0.10 | 10.55 | **0.006** |
|  | Community:Season:Condition | 2 | 7.30 | 0.05 | 2.54 | 0.106 |
|  | Residual | 23 | 33.10 | 0.23 |  |  |
|  | Total | 34 | 146.08 | 1.00 |  |  |
| **Hourly DON fluxes** | Community | 2 | 0.052 | 0.01 | 0.60 | 0.559 |
|  | Season | 1 | 0.093 | 0.02 | 2.12 | 0.155 |
|  | Condition | 1 | 0.000 | 0.00 | 0.00 | 0.998 |
|  | Community:Season | 2 | 0.222 | 0.05 | 2.53 | 0.105 |
|  | Community:Condition | 2 | 0.980 | 0.21 | 11.21 | **0.001** |
|  | Season:Condition | 1 | 1.797 | 0.38 | 41.12 | **0.001** |
|  | Community:Season:Condition | 2 | 0.533 | 0.11 | 6.10 | **0.014** |
|  | Residual | 23 | 1.005 | 0.21 |  |  |
|  | Total | 34 | 4.682 | 1.00 |  |  |
| **Daily DOC fluxes** | Community | 2 | 22408 | 0.36 | 36.83 | **0.001** |
|  | Season | 1 | 11463 | 0.18 | 37.68 | **0.001** |
|  | Community:Season | 2 | 14959 | 0.24 | 24.58 | **0.001** |
|  | Residual | 45 | 13.691 | 0.22 |  |  |
|  | Total | 50 | 62522 | 1.00 |  |  |
| **Daily DON fluxes** | Community | 2 | 77.45 | 0.09 | 4.09 | **0.028** |
|  | Season | 1 | 44.62 | 0.05 | 4.71 | **0.032** |
|  | Community:Season | 2 | 312.05 | 0.36 | 16.48 | **0.001** |
|  | Residual | 45 | 426.04 | 0.50 |  |  |
|  | Total | 50 | 860.15 | 1.00 |  |  |

**Table S5**. Adjusted p-values from multilevel pairwise comparisons of hourly DOC and DON fluxes between *Community* and *Condition*, and daily DOC and DON fluxes between *Community* and *Season*. The comparisons are performed using Tukey’s honest significant difference (HSD) test. Bold p-values indicate combinations that differ significantly (p < 0.05).

| **Hourly DOC fluxes** | Seagrass (daylight) | Seagrass  (dark) | Sponge  (daylight) | Sponge  (dark) | Association  (daylight) | Association  (dark) |
| --- | --- | --- | --- | --- | --- | --- |
| Seagrass (daylight) | x |  |  |  |  |  |
| Seagrass  (dark) | **0.010** | x |  |  |  |  |
| Sponge  (daylight) | **0.004** | 0.326 | x |  |  |  |
| Sponge  (dark) | **0.004** | 0.680 | 0.533 | x |  |  |
| Association  (daylight) | 0.204 | 0.110 | **0.038** | 0.105 | x |  |
| Association  (dark) | **0.010** | 0.389 | 0.873 | 0.624 | 0.051 | x |
| **Hourly DON fluxes** | Seagrass (daylight) | Seagrass  (dark) | Sponge  (daylight) | Sponge  (dark) | Association  (daylight) | Association  (dark) |
| Seagrass (daylight) | x |  |  |  |  |  |
| Seagrass  (dark) | 0.374 | x |  |  |  |  |
| Sponge  (daylight) | 0.240 | 0.609 | x |  |  |  |
| Sponge  (dark) | 0.812 | 0.085 | **0.027** | x |  |  |
| Association  (daylight) | 0.621 | 0.425 | 0.181 | 0.225 | x |  |
| Association  (dark) | 0.247 | 0.765 | 0.808 | 0.058 | 0.297 | x |
| **Daily DOC fluxes** | Seagrass (Autumn) | Seagrass  (Spring) | Sponge (Autumn) | Sponge  (Spring) | Association (Autumn) | Association  (Spring) |
| Seagrass (Autumn) | x |  |  |  |  |  |
| Seagrass (Spring) | **0.006** | x |  |  |  |  |
| Sponge (Autumn) | **0.006** | **0.002** | x |  |  |  |
| Sponge  (Spring) | **0.001** | **0.001** | **0.002** | x |  |  |
| Association (Autumn) | 0.924 | **0.049** | 0.056 | **0.001** | x |  |
| Association (Spring) | **0.001** | **0.001** | **0.001** | **0.011** | **0.002** | x |
| **Daily DON fluxes** | Seagrass (Autumn) | Seagrass  (Spring) | Sponge (Autumn) | Sponge  (Spring) | Association (Autumn) | Association  (Spring) |
| Seagrass (Autumn) | x |  |  |  |  |  |
| Seagrass (Spring) | **0.001** | x |  |  |  |  |
| Sponge (Autumn) | **0.001** | 0.206 | x |  |  |  |
| Sponge  (Spring) | 0.273 | **0.006** | **0.004** | x |  |  |
| Association (Autumn) | 0.335 | **0.036** | 0.080 | 0.137 | x |  |
| Association (Spring) | **0.004** | **0.001** | **0.001** | 0.559 | **0.014** | x |

**Table S6.** PERMANOVA for hourly (µmol g DW⁻¹ h⁻¹) and daily (µmol g DW⁻¹ d⁻¹) NH_4_^+^, NO_x_^-^, PO_4_^3-^ fluxes (µmol g DW⁻¹ h⁻¹) across different *Community* types, *Seasons*, *Condition* (light vs dark, when present) and their interaction terms. The table provides the degrees of freedom (Df), sum of squares (SS), proportion of variance explained (R²), pseudo-F statistics, and associated p-values (P(>F)) for each source of variation. Bold p-values (p < 0.05) indicate which factors contribute to differences in the measured variables.

| **Variable** | **Source of variation** | **Df** | **SS** | **R^2^** | **Pseudo-F** | **P (>F)** |
| --- | --- | --- | --- | --- | --- | --- |
| **Hourly NH_4_^+^ fluxes** | Community | 2 | 0.07 | 0.27 | 16.38 | **0.001** |
|  | Season | 1 | 0.00 | 0.02 | 1.93 | 0.178 |
|  | Condition | 1 | 0.01 | 0.05 | 6.44 | **0.026** |
|  | Community:Season | 2 | 0.04 | 0.16 | 9.86 | **0.003** |
|  | Community:Condition | 2 | 0.01 | 0.06 | 3.41 | **0.050** |
|  | Season:Condition | 1 | 0.04 | 0.18 | 21.09 | **0.001** |
|  | Community:Season:Condition | 2 | 0.01 | 0.03 | 1.58 | 0.233 |
|  | Residual | 28 | 0.06 | 0.23 |  |  |
|  | Total | 39 | 0.25 | 1.00 |  |  |
| **Hourly NO_x_^-^ fluxes** | Community | 2 | 3.27 | 0.56 | 31.06 | **0.001** |
|  | Season | 1 | 0.06 | 0.01 | 1.22 | 0.298 |
|  | Condition | 1 | 0.36 | 0.06 | 6.76 | **0.009** |
|  | Community:Season | 2 | 0.08 | 0.01 | 0.72 | 0.509 |
|  | Community:Condition | 2 | 0.52 | 0.09 | 4.91 | **0.013** |
|  | Season:Condition | 1 | 0.05 | 0.01 | 0.96 | 0.347 |
|  | Community:Season:Condition | 2 | 0.01 | 0.00 | 0.13 | 0.872 |
|  | Residual | 28 | 1.47 | 0.25 |  |  |
|  | Total | 39 | 5.82 | 1.00 |  |  |
| **Hourly PO_4_^3-^ fluxes** | Community | 2 | 0.001 | 0.29 | 9.24 | **0.002** |
|  | Season | 1 | 0.000 | 0.08 | 4.93 | **0.040** |
|  | Condition | 1 | 0.000 | 0.00 | 0.00 | 0.980 |
|  | Community:Season | 2 | 0.000 | 0.04 | 1.13 | 0.328 |
|  | Community:Condition | 2 | 0.000 | 0.05 | 1.62 | 0.204 |
|  | Season:Condition | 1 | 0.000 | 0.00 | 0.06 | 0.797 |
|  | Community:Season:Condition | 2 | 0.000 | 0.09 | 2.86 | 0.086 |
|  | Residual | 28 | 0.001 | 0.45 |  |  |
|  | Total | 39 | 0.002 | 1.00 |  |  |
| **Daily NH_4_^+^ fluxes** | Community | 2 | 79.80 | 0.58 | 86.21 | **0.001** |
|  | Season | 1 | 0.89 | 0.01 | 1.92 | 0.189 |
|  | Community:Season | 2 | 30.60 | 0.22 | 33.06 | **0.001** |
|  | Residual | 58 | 26.84 | 0.19 |  |  |
|  | Total | 63 | 138.13 | 1.00 |  |  |
| **Daily NO_x_^-^ fluxes** | Community | 2 | 2763.4 | 0.78 | 121.04 | **0.001** |
|  | Season | 1 | 50.6 | 0.01 | 4.43 | **0.048** |
|  | Community:Season | 2 | 53.8 | 0.02 | 2.35 | 0.116 |
|  | Residual | 58 | 662.1 | 0.19 |  |  |
|  | Total | 63 | 3529.8 | 1.00 |  |  |
| **Daily PO_4_^3-^ fluxes** | Community | 2 | 0.77 | 0.48 | 37.66 | **0.001** |
|  | Season | 1 | 0.23 | 0.14 | 21.96 | **0.001** |
|  | Community:Season | 2 | 0.03 | 0.02 | 1.59 | 0.186 |
|  | Residual | 58 | 0.60 | 0.37 |  |  |
|  | Total | 63 | 1.63 | 1.00 |  |  |

**Table S7**. Adjusted p-values from multilevel pairwise comparisons of hourly NH_4_^+^ and NO_x_^-^ fluxes between *Community* and *Condition*, and daily NH_4_^+^ fluxes between *Community* and *Season*. The comparisons are performed using Tukey’s honest significant difference (HSD) test. Bold p-values indicate combinations that differ significantly (p < 0.05).

| **Hourly NH_4_^+^ fluxes** | Seagrass (daylight) | Seagrass  (dark) | Sponge  (daylight) | Sponge  (dark) | Association  (daylight) | Association  (dark) |
| --- | --- | --- | --- | --- | --- | --- |
| Seagrass (daylight) | x |  |  |  |  |  |
| Seagrass  (dark) | 0.427 | x |  |  |  |  |
| Sponge  (daylight) | **0.023** | **0.002** | x |  |  |  |
| Sponge  (dark) | 0.359 | **0.019** | **0.035** | x |  |  |
| Association  (daylight) | 0.285 | **0.018** | 0.110 | 0.802 | x |  |
| Association  (dark) | 0.197 | **0.005** | 0.059 | 0.628 | 0.886 | x |
| **Hourly NO_x_^-^ fluxes** | Seagrass (daylight) | Seagrass  (dark) | Sponge  (daylight) | Sponge  (dark) | Association  (daylight) | Association  (dark) |
| Seagrass (daylight) | x |  |  |  |  |  |
| Seagrass  (dark) | 0.051 | x |  |  |  |  |
| Sponge  (daylight) | **0.002** | **0.005** | x |  |  |  |
| Sponge  (dark) | **0.001** | **0.004** | **0.037** | x |  |  |
| Association  (daylight) | **0.005** | **0.004** | **0.038** | **0.002** | x |  |
| Association  (dark) | **0.002** | **0.001** | 0.228 | **0.002** | 0.192 | x |
| **Daily NH_4_^+^ fluxes** | Seagrass (Autumn) | Seagrass  (Spring) | Sponge (Autumn) | Sponge  (Spring) | Association (Autumn) | Association  (Spring) |
| Seagrass (Autumn) | x |  |  |  |  |  |
| Seagrass (Spring) | **0.001** | x |  |  |  |  |
| Sponge (Autumn) | **0.002** | **0.001** | x |  |  |  |
| Sponge  (Spring) | **0.002** | **0.001** | **0.001** | x |  |  |
| Association (Autumn) | **0.001** | **0.001** | **0.009** | **0.027** | x |  |
| Association (Spring) | 0.105 | **0.001** | 0.368 | **0.001** | **0.005** | x |

**Table S8.** PERMANOVA for δ¹³C and δ¹⁵N values and C:N ratios across different *Sample* types and *Association* types. The table provides the degrees of freedom (Df), sum of squares (SS), proportion of variance explained (R²), pseudo-F statistics, and associated p-values (P(>F)) for each source of variation. Bold p-values (p < 0.05) indicate which factors contribute to differences in the measured variables.

| **Variable** | **Source of variation** | **Df** | **SS** | **R^2^** | **Pseudo-F** | **P (>F)** |  |
| --- | --- | --- | --- | --- | --- | --- | --- |
| **δ¹³C values** | Sample | 2 | 658.12 | 0.82 | 208.75 | **0.001** |  |
|  | Association | 1 | 2.35 | 0.00 | 1.49 | 0.236 |  |
|  | Sample:Association | 2 | 1.15 | 0.00 | 0.37 | 0.700 |  |
|  | Residual | 91 | 143.45 | 0.18 |  |  |  |
|  | Total | 96 | 805.07 | 1.00 |  |  |  |
| **δ¹⁵N values** | Sample | 2 | 75.99 | 0.46 | 50.64 | **0.001** |  |
|  | Association | 1 | 13.83 | 0.08 | 18.44 | **0.001** |  |
|  | Sample:Association | 2 | 7.56 | 0.05 | 5.04 | **0.005** |  |
|  | Residual | 91 | 68.28 | 0.41 |  |  |  |
|  | Total | 96 | 165.66 | 1.00 |  |  |  |
| **C:N ratios** | | Sample | 2 | 3241.8 | 0.71 | 112.31 | **0.001** |
|  |  | Association | 1 | 6.7 | 0.00 | 0.46 | 0.509 |
|  |  | Sample:Association | 2 | 9.4 | 0.00 | 0.32 | 0.692 |
|  |  | Residual | 91 | 1313.4 | 0.29 |  |  |
|  |  | Total | 96 | 4571.3 | 1.00 |  |  |

**Table S9**. Adjusted p-values from multilevel pairwise comparisons of δ¹⁵N values between *Sample* types and *Association* types. The comparisons are performed using Tukey’s honest significant difference (HSD) test. Bold p-values indicate combinations that differ significantly (p < 0.05).

| **δ^15^N values** | *P. oceanica* leaves  (associated) | *P. oceanica* leaves  (not associated) | *C. nucula*  (associated) | *C. nucula*  (not associated) | *P. oceanica* epiphytes  (associated) | *P. oceanica* epiphytes  (not associated) |
| --- | --- | --- | --- | --- | --- | --- |
| *P. oceanica* leaves (associated) | x |  |  |  |  |  |
| *P. oceanica* leaves  (not associated) | **0.001** | x |  |  |  |  |
| *C. nucula* (associated) | **0.001** | **0.001** | x |  |  |  |
| *C. nucula*  (not associated) | **0.001** | **0.001** | 0.734 | x |  |  |
| *P. oceanica* epiphytes (associated) | **0.001** | **0.001** | 0.106 | 0.104 | x |  |
| *P. oceanica* epiphytes  (not associated) | 0.064 | **0.001** | **0.011** | **0.029** | **0.027** | x |
